# Electrostatically Driven Encapsulation of Hydrophilic, Non-Conformational Peptide Epitopes into Liposomes

**DOI:** 10.3390/pharmaceutics11110619

**Published:** 2019-11-18

**Authors:** Ehsan Suleiman, Dominik Damm, Mirjam Batzoni, Vladimir Temchura, Andreas Wagner, Klaus Überla, Karola Vorauer-Uhl

**Affiliations:** 1 Polymun Scientific Immunbiologische Forschung GmbH, 3400 Klosterneuburg, Austria; 2 Department of Biotechnology, University of Natural Resources and Life Sciences, 1190 Vienna, Austria; 3 Institute of Clinical and Molecular Virology, Universitätsklinikum Erlangen, 91054 Erlangen, Germany; 4 FH Campus Wien - University of Applied Sciences, 1100 Vienna, Austria

**Keywords:** electrostatic interactions, encapsulation, encapsulation efficiency, hydrophilic peptides, in situ binding, liposomes, microfluidics, peptides, peptide-membrane interaction, vaccines

## Abstract

Since the first use of liposomes as carriers for antigens, much work has been done to elucidate the mechanisms involved in the encapsulation of vaccine-relevant biomolecules. However, only a few studies have specifically investigated the encapsulation of hydrophilic, non-conformational peptide epitopes. We performed comprehensive and systematic screening studies, in order to identify conditions that favor the electrostatic interaction of such peptides with lipid membranes. Moreover, we have explored bi-terminal sequence extension as an approach to modify the isoelectric point of peptides, in order to modulate their membrane binding behavior and eventually shift/expand the working range under which they can be efficiently encapsulated in an electrostatically driven manner. The findings of our membrane interaction studies were then applied to preparing peptide-loaded liposomes. Our results show that the magnitude of membrane binding observed in our exploratory in situ setup translates to corresponding levels of encapsulation efficiency in both of the two most commonly employed methods for the preparation of liposomes, i.e., thin-film hydration and microfluidic mixing. We believe that the methods and findings described in the present studies will be of use to a wide audience and can be applied to address the ongoing relevant issue of the efficient encapsulation of hydrophilic biomolecules.

## 1. Introduction

Peptides are widely acknowledged as being unique and indispensable pharmaceutical compounds. A plethora of peptide therapeutics and peptide vaccines, both prophylactic and therapeutic, have been evaluated in clinical trials. Many more are currently undergoing clinical testing for a broad range of indications, predominantly in the therapeutic areas of oncologic, metabolic and cardiovascular diseases. While more than 60 therapeutic peptides have been approved for human use, not a single peptide-based vaccine has been licensed so far [1,2]. Challenges with regard to their stability, bioavailability and immunogenicity have been successfully addressed by a multitude of strategies such as the use of particulate delivery systems or the administration of peptide epitopes as part of rationally designed multi-epitope fusion proteins, polypeptides or bioconjugates [3,4]. Liposomes and other lipid-based delivery systems are of particular interest, as their composition and physical properties can be adapted to meet the requirements of particular applications, e.g., lymph node or dendritic cell targeting [5]. Moreover, the use of liposomes allows the co-delivery of adjuvants such as MPLA, CpG ODN and poly I:C [6,7]. Importantly, various scalable methods that enable the industrial manufacturing of clinical-grade material are available [8,9].

Nevertheless, the controlled and efficient encapsulation of peptides in the context of vaccine manufacturing remains a demanding task. This is partly because peptides display a large diversity with regard to their aqueous solubility, hydropathicity and isoelectric point, i.e., net charge/pH profile [10]. In agreement with theoretical considerations on the electrostatic binding of peptides to lipid membranes, experimental work on protein encapsulation has identified ionic strength, pH, the isoelectric point (pI) and the liposome composition (i.e., addition of charged membrane components), as the main determinants of a predominantly electrostatically driven encapsulation of hydrophilic proteins [11,12,13,14,15,16,17]. Interestingly, molecular dynamics simulations suggest that liposome size, protein size and the speed of liposome formation contribute negligibly to the overall levels of encapsulation efficiency [18]. On the contrary, purely passive encapsulation is considered to be largely determined by the entrapped volume, which in turn is known to be a function of the liposome size and the total lipid concentration [19]. Notably, recent microfluidics studies on the passive (non-electrostatic) encapsulation of ovalbumin suggest that encapsulation efficiency is largely determined by process parameters such as the total flow rate and the flow rate ratio. Moreover, these studies indicate that the alkyl chain lengths of the employed phosphatidylcholines have no significant impact on the encapsulation of ovalbumin into neutrally charged liposomes [20].

As the implementation of these findings is likely to result in increased controllability in the process of peptide encapsulation, we decided to further investigate these findings within the context of our ongoing efforts to develop a liposome-based vaccine designed to harness intrastructural help [21]. These liposomes carry T helper cell peptides (i.e., non-conformational peptide epitopes) within their aqueous interior, display an immunogen on their surface, and are referred to as T helper liposomes. Recently published research on virus-like particle vaccines against HIV-1 has provided a compelling proof of concept for this type of strategy [22,23]. Moreover, independent reports on liposome-based malaria and group A streptococci vaccines utilizing the same principle are highly encouraging [24,25].

Here, we investigated the N- and C-terminal introduction of charged amino acids in order to expand the physico-chemical conditions under which hydrophilic, non-conformational peptide epitopes can be efficiently encapsulated in an electrostatically driven manner. OVA 323-339, a widely used model peptide, were selected for the purpose of systematically investigating this approach in a model system comprising cationic peptides and anionic liposomes. Variants with elevated positive net charge were realized by bi-terminally extending the sequence with increasing numbers of lysine residues. Lysine was selected over arginine as it displays a lower helix-forming propensity [26]. A random coiled conformation is favored over an ɑ-helical one, as it offers potentially increased electrostatic controllability over the interaction between peptides and lipid membranes. This is especially true for amphipathic ɑ-helical peptides, which may contribute a distinct hydrophobic component to the interactions. Moreover, it has been reported that polyarginine binds to pure phosphatidylcholine membranes, which limits the usefulness of arginine for the intended sequence extension [27]. In contrast, the binding of polylysine to lipid membranes requires the presence of a negatively charged membrane component, e.g., phosphatidylglycerol (PG). Interestingly, polylysine exhibits strong anticooperative binding behavior, which adds an additional layer of electrostatic controllability. Altogether, these findings render lysine an ideal basic amino acid for modifying peptides in order to investigate the proposed encapsulation approach. Bi-terminal flanking was preferred over sole N- or C-terminal extension, as it has been suggested that this may reduce possible self-oligomerization and aggregation of peptides upon binding to lipid membranes [28].

Circular dichroism (CD) spectroscopic studies, as well as zeta-potential and particle size measurements, were employed to study peptide-membrane interactions as a function of various physico-chemical properties, membrane compositions and peptide sequences [29,30,31]. Binding studies and encapsulation experiments using the two most commonly employed methods for the lab-scale preparation of peptide-loaded liposomes (i.e., thin-film hydration and microfluidic mixing), were then used to test whether the extent of electrostatic binding translates to corresponding levels of encapsulation. Encapsulation experiments were performed with anionic, cationic and functionalized liposomes, i.e., liposomes that carry functionalized lipids for the coupling of immunogens. Finally, a set of in vitro experiments was performed to investigate the effect of lysine flanking on peptide loading by MHC class II molecules (MHC-II) and the recognition of MHC-II/peptide complexes by respective antigen-specific CD4^+^ T cells.

To the best of our knowledge this is the first systematic investigation of bi-terminal lysine flanking as a strategy for modulating the electrostatically driven encapsulation of hydrophilic, non-conformational peptide epitopes into liposomes.

## 2. Materials and Methods

### 2.1. Materials

1,2-dioleoyl-sn-glycero-3-phosphocholine (DOPC), 1,2-distearoyl-sn-glycero-3-phosphocholine (DSPC), 1,2-dioleoyl-sn-glycero-3-phospho-(1’-rac-glycerol) (sodium salt) (DOPG) and 1,2-distearoyl-sn-glycero-3-phospho-(1’-rac-glycerol) (sodium salt) (DSPG) were purchased from Lipoid GmbH, Germany. (3β)-cholest-5-en-3-ol (cholesterol) was purchased from Dishman Netherlands B.V., Veenendaal, Netherlands. 1,2-dioleoyl-sn-glycero-3-[(*N*-(5-amino-1-carboxypentyl) iminodiacetic acid)succinyl] (nickel salt) (18:1 DGS-NTA(Ni)) and 1,2-dipalmitoyl-sn-glycero-3-phosphoethanolamine-*N*-(dodecanoyl) (sodium salt) (16:0 Dodecanoyl PE) were purchased from Avanti Polar Lipids, Inc., Alabaster, AL, USA. 1,2-dioleoyl-3-trimethylammonium-propane (chloride salt) (DOTAP) was purchased from Merck KGaA, Darmstadt, Germany. Monodisperse 1,2-distearoyl-sn-glycero-3-phosphoethanolamine-*N*-[carboxy(polyethylene glycol)] with 14 ethylene glycol units (DSPE-PEG14-COOH) was custom-synthesized with a purity of ≥ 95% (Biochempeg Scientific Inc., Watertown, MA, USA). All other lipids were of ≥ 98% purity. Methanol, chloroform and ethanol were of Reag. Ph. Eur. quality and were both obtained from Merck KGaA, Darmstadt, Germany. OVA 323-339, with a purity of ≥ 98%, was obtained from piCHEM, Grambach, Austria. The three lysine-flanked peptide variants OVA 323-339 1K, OVA 323-339 2K and OVA 323-339 3K were custom-synthesized with a purity of ≥ 95% (ProteoGenix, Schiltigheim, France). All peptides were synthesized as TFA salts. All chemicals used to prepare the buffers were of analytical grade or of Ph. Eur. quality and were obtained from Merck KGaA, Darmstadt, Germany. An Ultra Clear UV unit (SG Wasseraufbereitung und Regenerierstation GmbH, Barsbüttel, Germany) provided the purified water (18.2 MΩ at 25 °C) used in this study.

### 2.2. Buffers

Acetate buffers (5 mM ABS pH 4.0 and 5 mM AB sucrose pH 4.0) were composed of 4.1 mM CH_3_COOH and 0.9 mM Na(CH_3_COO). Phosphate buffers (5 mM PBS pH 6.5, 5 mM PB sucrose pH 6.5) were composed of 1.5 mM Na_2_HPO_4_·2H_2_O and 3.5 mM KH_2_PO_4_. Borate buffers (5 mM BBS pH 8.5 and 5 mM BB sucrose pH 8.5) were composed of 1 mM Na_2_B_4_O_7_ 10 H_2_O and 4 mM H_3_BO_3_. Buffers referred to as having a high ionic strength (5 mM ABS pH 4.0, 5 mM PBS pH 6.5, etc.) contain 150 mM sodium chloride, while those referred to as having a low ionic strength (5 mM AB sucrose pH 4.0, 5 mM PB sucrose pH 6.5, etc.) contain 300 mM sucrose instead. Buffers were routinely analyzed for pH, conductivity and osmolality.The osmolality of the sodium chloride-containing buffers (i.e., high-ionic-strength buffers with a conductivity of ≥ 12 mS/cm) was around 280 mOsm/kg while that of the sucrose-containing buffers (i.e., low-ionic-strength buffers with a conductivity of ≤ 2 mS/cm) was around 340 mOsm/kg. All buffers were filtered through a 0.22-µm filter prior to use.

### 2.3. Liposome Preparation

#### 2.3.1. Liposome Composition

Liposomes prepared as part of the investigation into electrostatically driven peptide encapsulation were predominantly composed of dioleoyl lipids. Anionic liposomes were composed of 85 mol% DOPC and 15 mol% DOPG, whereas cationic liposomes were composed of 85 mol% DOPC and 15 mol% DOTAP. Neutral liposomes were composed of 100 mol% DOPC. Charged functionalized liposomes were composed of 81 mol% DOPC, 15 mol% charged lipid (DOPG or DOTAP) and 4 mol% functionalized lipids (18:1 DGS-NTA(Ni), 16:0 Dodecanoyl PE or DSPE-PEG14-COOH). Liposomes used in the interaction studies and in the in vitro experiments were prepared from distearoyl lipids and cholesterol. Anionic liposomes were composed of 45 mol% DSPC, 40 mol% cholesterol and 15 mol% DSPG, whereas neutral liposomes were composed of 60 mol% DSPC and 40 mol% cholesterol.

#### 2.3.2. Thin-Film Hydration

The desired quantities of lipids were dissolved in an appropriate volume of chloroform/methanol (2:1, by volume) and transferred to a glass vial. The solvent was evaporated overnight at room temperature and atmospheric pressure. Alternatively, lipids were dissolved in an appropriate volume of chloroform/methanol (2:1, by volume) and transferred into a round-bottomed flask. For evaporation of the solvent, the water bath of a rotary evaporator was set to 55 °C. Rotation was set to 150 rpm. The evacuation was performed stepwise: five minutes (min) at atmospheric pressure, 15 min at 450 mbar and 10 min at < 40 mbar. The latter lipid-film generation approach was only used for medium-scale preparations (> 2.0 mL preparation volume) and for formulations that predominantly contained distearoyl lipids. Subsequently, the film was flush-dried with nitrogen. Hydration of the lipid film with buffer or peptide solutions of different concentrations, as well as the subsequent downsizing by means of extrusion, were performed at elevated temperatures. Extrusion was performed on a LIPEX™ Extruder (Northern Lipids Inc., Burnaby, BC, Canada) equipped with Whatman^®^ Nuclepore track-etched membranes. Up to five extrusion cycles with 400-nm membranes and an additional five to ten cycles with 200-nm and/or 100-nm membranes, were performed. While formulations that contained low-phase-transition-temperature lipids were processed at 35 °C, those that contained high-phase-transition-temperature lipids were processed at 55 °C. The resulting liposome suspension was filtered (0.2 µm) using a Supor^®^ Acrodisc^®^ syringe filter and stored at 2–8 °C until further analysis.

#### 2.3.3. Microfluidic Mixing

Preparation of liposomes via microfluidic mixing was performed at room temperature using a polycarbonate herringbone mixer (microfluidic ChipShop GmbH, Jena, Germany). A Qmix system equipped with two neMESYS mid-pressure syringe pumps (CETONI GmbH, Korbußen, Germany) was used to mix the lipid solution (55 mM in ethanol, 96% (*v*/*v*), 0.15 mL, 0.25 mL/min) with the peptide solution (24.75 µM in buffer, 0.5 mL, 0.83 mL/min). The resulting liposome suspension was then further diluted with 1 mL of buffer, sterile-filtered and stored at 2–8 °C until further analysis.

#### 2.3.4. Tangential Flow Filtration (TFF)

Non-encapsulated peptide was removed by TFF using a 100 kDa mPES MicroKros^®^ module (Repligen, Waltham, MA, USA) with a surface area of 20 cm² [20]. Up to ten cycles of filtration (volume exchanges) using an electrostatic binding-impeding high-ionic-strength buffer (e.g., 5 mM PBS pH 6.5 or 5 mM ABS pH 4.0) and then an additional five to ten cycles using a low-ionic-strength buffer (e.g., 5 mM PB Sucrose pH 6.5 or 5 mM AB Sucrose pH 4.0) were performed, to remove non-encapsulated (i.e., both free and outer surface-associated) peptide.

### 2.4. Particle Size and Zeta Potential

A Zetasizer Nano ZS (Malvern Instruments Ltd., Malvern, United Kingdom) was used to perform the particle size analysis and measure the zeta potentials. Measurements were performed in triplicate at 25 °C. Measurements to study the interactions between peptides and liposomes were performed at a constant lipid concentration of 200 µM or 400 µM. For this purpose, liposomes (w/o peptide) were diluted with appropriate buffers and mixed with peptide to give molar peptide:lipid ratios of 1:4, 1:12, 1:36, 1:108, 1:133 and 1:324. Relevant buffer properties, such as viscosity or permittivity, were calculated using the complex solvent builder add-in (Zetasizer Nano ZS software, version 7.03).

### 2.5. CD Spectroscopy

CD spectroscopy was performed on a Chirascan system (Applied Photophysics Ltd., Leatherhead, United Kingdom) in the far-UV region (190-260 nm) at room temperature. Measurements were performed at a peptide concentration of 20 μM. The path length of the cuvette was 1 mm. Bandwidth and step size were both set to 1 nm and the scan time per point was set to 10 s.

### 2.6. HPLC

#### 2.6.1. General

Quantifications were performed on a 1200 series Agilent Technologies HPLC system (Agilent Technologies, Santa Clara, CA, USA). The system was operated via ChemStation (Rev. B.04.01 SP1 [647]) and was equipped with a degasser, a binary pump, a column oven, an autosampler, a diode array detector (DAD) and a SEDEX Model 85 LT-ELSD (low-temperature evaporative light scattering detector) (SEDERE, Olivet, France). Both the peptide and lipid quantifications were performed on a Luna^®^ 5 µm C18 100 Å (150 × 4.6 mm) system from Phenomenex Inc., Torrance, CA, USA. HPLC solvents were of LiChrosolv^®^ gradient grade and were obtained from Merck KGaA, Darmstadt, Germany. Trifluoroacetic acid (TFA) for synthesis with a purity of ≥ 98% was also from Merck KGaA.

#### 2.6.2. Peptide Quantification

The column oven and the autosampler chamber were both set to 20 °C. The injection volume was set to 50 µL or 100 µL. While lipid-containing samples were subjected to peptide extraction prior analysis, samples in aqueous lipid-free matrices did not require any particular sample preparation. The detection of the peptides was performed on a diode array detector at 202 nm (bandwidth: 4 nm). Samples extracted from cell culture media were detected at 202 nm (bandwidth: 4 nm), using a reference wavelength of 226 nm (bandwidth: 1 nm) to account for the interfering peak of an unknown compound of the medium. A gradient of 100% (*v*/*v*) water + 0.1% (*v/v*) TFA (mobile phase A) and 100% acetonitrile + 0.1% (*v*/*v*) TFA (mobile phase B) was run at a flow rate of 1 mL/min for a total run time of 18.5 min. The gradient was set up as follows: 5–30% B in 12.5 min, 30–100% B in 0.5 min, 100% B for 2.5 min, 100–5% B in 0.5 min and 5% B for 2.5 min. Further details (e.g., on method validation and calibration) are summarized in the Supplementary Material (Table S1).

#### 2.6.3. Peptide Extraction

The protocol for the extraction of peptides from liposomes or a lipid-containing matrix was adapted from that of Penwell and colleagues, with minor modifications [32]. Peptide extraction was performed in order to determine the total (i.e., encapsulated plus non-encapsulated) peptide concentration from liposomes. Briefly, 135 µL of the sample were mixed with 15 µL of 0.8 M sodium bicarbonate solution in a regular 1.5-mL polypropylene centrifugation tube. 150 µL of water-saturated n-butanol was then added to extract the lipids. Extraction was performed in an Eppendorf ThermoMixer^®^ at 1400 rpm for 15 min. Centrifugation at 3000 × g was performed for 10 min to separate the phases. The aqueous phase on the bottom was collected and stored at −20 °C until further analysis. The minor but consistent increase in volume of the aqueous phase as a result of the extraction procedure was corrected using a correction factor of 1.08. Verification of the extraction procedure revealed an extraction efficiency in the range 91.6–102.2% for all peptide variants used in this study. Samples without lipids (liposomes) as well as samples with a molar peptide:lipid ratio of 1:67.5 and 1:135, were included in the method verification. Extraction efficiency was independent of the peptide variant and the tested molar peptide:lipid ratios.

#### 2.6.4. Lipid Quantification

The column oven was set to 35 °C, whereas the autosampler chamber was set to 25 °C. Samples were diluted with isopropanol to give a clear solution, with a final isopropanol content of 70% (*v*/*v*). Predilutions were made with purified water. The injection volume was 50 µL. Detection of DSPC, DSPG and cholesterol was performed on a gain-setting-adjustable ELSD. It is worth noting, although it is not of relevance to the present investigation, that this method can also be used to quantify corresponding fatty acids and lysophospholipids. Moreover, this method can be used to simultaneously quantify peptides from neutral liposomal formulations without prior extraction. The gradients of mobile phase A (9% (*v*/*v*) water + 5% (*v*/*v*) methanol + 0.0325% (*v*/*v*) TFA) and mobile phase B (95% (*v*/*v*) isopropanol + 5% (*v*/*v*) methanol + 0.125% (*v*/*v*) TFA) were run at a flow rate of 0.875 mL/min for a total runtime of 40.5 min. The gradient was set up as follows: 0% B for 2.5 min, 0–20% B in 7.5 min, 20–70% B in 3.0 min, 70–90% B in 22.0 min, 90% B for 2.5 min, 90–0% B in 0.5 min and 0% B for 2.5 min. Further details (e.g., on method validation and calibration) can be found in the supplement (Table S2).

### 2.7. Peptide Binding

Liposomes (w/o peptide) were diluted with appropriate buffers and mixed with peptide to give molar peptide:lipid ratios of 1:4, 1:12, 1:36, 1:108 and 1:324, at a constant peptide concentration of 43 µM. Unbound peptide was separated using Centrisart I^®^ centrifugal ultrafiltration units with a cut-off of 100 kDa. These units were used according to the manufacturer’s instructions. Both the initial mixture of liposomes and peptide and the collected ultrafiltrate, were quantified by HPLC. The fraction of liposome-associated (bound) peptide was indirectly calculated on the basis of these HPLC results. The percentage of unspecific binding of OVA 323-339, OVA 323-339 1K and OVA 323-339 2K to the polyethersulfone membrane of the ultrafiltration unit under the relevant experimental settings, was determined to be 5.9 ± 4.6%, 12.2 ± 3.0% and 14.5 ± 5.1% respectively. Please note that although this unspecific binding might lead to a general overestimation of the peptide binding to the liposomes, we decided not to use any post hoc corrections, as there were no relevant or statistically significant differences in the extent of unspecific binding among the peptide variants used in the respective experiments.

### 2.8. Encapsulation Efficiency

The encapsulation efficiency of the liposomes used in the in vitro studies was calculated on the basis of the peptide concentration after hydration of the lipid film and that after the removal of non-encapsulated peptide by means of TFF. The peptide concentration at the level of film hydration was calculated on the basis of the initially added amount of peptide. In contrast, purified liposomes were subjected to extraction and their peptide content was determined by HPLC. Likewise, the lipid content was determined, in order to take into account processing-associated losses (i.e., losses associated with extrusion, sterile filtration, etc.) and changes in concentration as a result of the consecutive cycles of dilution and concentration during TFF. The average lipid recovery of the purified liposomal suspension was determined to be 87.9 ± 12.2%. An overview on the results of the lipid analysis can be found in the supplement (Table S9).

The encapsulation efficiency of all other liposomes that were prepared at a small scale (≤ 1.5 mL), was indirectly determined using Centrisart I^®^ centrifugal ultrafiltration units with a cut-off of 100 kDa. Here, encapsulation efficiency was determined on the basis of the total and unbound peptide concentration immediately after extrusion. The peptide concentration of the liposomal suspension and that of the filtrate were both determined by HPLC. Clearly, calculating the encapsulation efficiency by this indirect method requires the non-encapsulated peptide to completely detach from the outer surface of the liposomes [33]. To ensure complete detachment of the peptides and to adjust the sample matrix for subsequent quantification via HPLC, samples were diluted with 1 M NaCl or 2 M sucrose, to give final NaCl and sucrose concentrations of 131 mM and 262 mM respectively. As part of a comprehensive method verification and in order to further corroborate the capability of our high-ionic-strength TFF setup to efficiently remove non-encapsulated peptide, we investigated whether these ionic-strength conditions are in fact sufficient to reverse peptide binding, i.e., to induce detachment of non-encapsulated but outer surface-associated peptide. For this purpose non-functionalized anionic liposomes (in a low-ionic-strength buffer with a pH of 6.5) were mixed with peptide to give a molar peptide:lipid ratio of 1:324. Sodium chloride-induced detachment of OVA 323-339, OVA 323-339 1K, OVA 323-339 2K and OVA 323-339 3K was found to occur with efficiencies of 97.3 ± 3.7%, 109.1 ± 10.9%, 97.8 ± 11.3% and 103.5 ± 8.1%, respectively. The percentages of unspecific binding of the peptide variants to the ultrafiltration membrane under the experimental settings relevant to the investigation of peptide encapsulation, were determined to be 8.1 ± 1.9%, 29.0 ± 1.2%, 33.4 ± 4.7% and 36.1 ± 4.7% respectively. Statistical analysis showed that the extent of unspecific binding among the lysine-flanked variants is comparable (not statistically different). In contrast, unspecific binding of OVA 323-339 is statistically significantly lower than that of its sequence-extended variants. Corrections (e.g., normalization to an unspecific binding of 0%), in order to enable a meaningful quantitative comparison of the encapsulation efficiency of OVA 323-339 with that of its modified variants are made explicit at relevant passages/graphs in this article. Although we refrain from making any strong generalized claims on the overall level of encapsulation efficiency, we wish to emphasize that this unspecific binding might lead to an overestimation of peptide encapsulation.

### 2.9. In Vitro Stability Study

Peptide-loaded liposomes were diluted with R10 medium [RPMI 1640 (Gibco™) supplemented with 10% FCS, 1% penicillin + streptomycin, 10 mM HEPES, 2 mM L-glutamine and 50 µM β-mercaptoethanol] to give a peptide concentration of 26 µM. The peptide-loaded liposomes were then incubated under cell culture conditions (37 °C, 5% CO2 and 70% relative humidity) for a period of 72 h without shaking. Samples were taken at 0, 24, 48 and 72 h, subjected to extraction and quantified for their peptide content. In a control experiment, peptides were mixed with empty liposomes (i.e., liposomes without any encapsulated peptides) and tested in the same manner.

### 2.10. Mice

Six- to eight-week-old C57BL/6NRj mice (Janvier Labs, Le Genest-Saint-Isle, France) and OT-II TCR transgenic mice (in-house breeding) were used. Mice were housed at the animal facility of the Franz Penzoldt Centre at the Friedrich–Alexander University Erlangen–Nürnberg in accordance with the national law (project identification code: TS-6/2015 Virologie, date of approval: 29 May 2015). Mice were handled according to the guidelines of the Federation of European Laboratory Animal Science Associations.

### 2.11. Cell Isolation and Purification

For the purification of DCs, single-cell suspensions of C57BL/6NRj mice splenocytes were prepared as previously described [34]. Briefly, CD11c^+^ DCs were enriched by positive selection with anti-CD11c magnetic beads (Miltenyi Biotec, Bergisch Gladbach, Germany). CD4^+^ T cells were isolated from a single-cell suspension of splenocytes from naïve OT-II mice using a CD4^+^ T-Cell Isolation Kit (Miltenyi Biotec, Bergisch Gladbach, Germany). All isolations were performed according to the manufacturer’s instructions.

### 2.12. CD4^+^ T-Cell Proliferation Assay

The proliferation assay for OT-II-specific CD4^+^ T cells in co-culture with splenic DCs from C57BL/6NRj mice, was performed as described elsewhere [35]. Briefly, CD4^+^ T cells were labelled with a CellTrace™ CFSE Cell Proliferation Kit from Thermo Fisher Scientific, Waltham, USA. The concentration of CFSE for labelling the cells was 7.5 µM. Labelled CD4^+^ T cells were then co-cultivated with freshly isolated splenic DCs for 64 h in the presence of liposomal or free (non-liposomal) peptide, as described in the respective figure captions. Cells were seeded in U-bottom 96-well plates in R10 medium at a density of 1 × 10^5^ CD4^+^ T cells and 1 × 10^5^ DCs per well. The proliferation assay was performed under standard cell culture conditions (37 °C, 5% CO_2_ and 70% relative humidity). The proliferation-dependent decrease of the CFSE fluorescence intensity of the labelled CD4^+^ T cells was analyzed on an LSR II flow cytometer (Becton, Dickinson and Company, Franklin Lakes, NJ, USA). CD4^+^ T-cell survival was determined by staining with Fixable Viability Dye (Thermo Fisher Scientific, Waltham, MA, USA).

## 3. Results and Discussion

### 3.1. Peptide Design and Characterization

To explore N- and C-terminal sequence extension as an approach to enabling and improving the controlled electrostatically driven encapsulation of hydrophilic peptides, three variants of the model T helper cell peptide OVA 323-339, with increasing numbers of flanked lysine residues, were custom-synthesized (Table 1).

**Figure 1:**
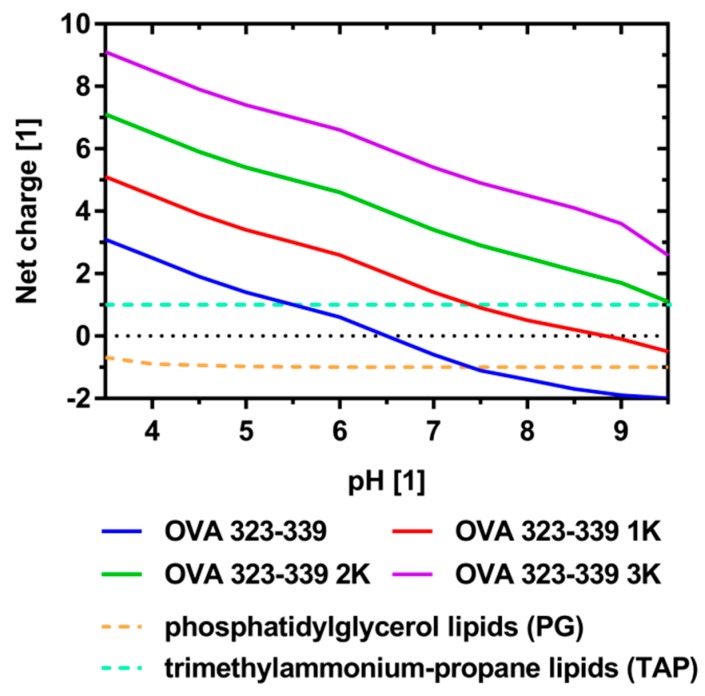
Net charge/pH profiles of the model peptides and study-relevant phospholipids. The net charge of the model peptides as a function of pH as calculated by PROTEIN CALCULATOR v3.4 is shown [37]. Data for the PG net charge/pH profile were adapted from [38]. TAP lipids such as DOTAP exhibit a pH-independent net charge of +1 [39].

To estimate the impact of these flanking residues on the hydropathicity, isoelectric point (pI) and the net charge at the pH values selected for encapsulation (pH 4.0, 6.5 and 8.5), sequences were analyzed in silico using various tools [36,37]. The grand average of hydropathicity (GRAVY) decreased as a result of the addition of lysine residues, suggesting a reduction in the relative contribution of hydrophobic interactions to the process of electrostatically driven peptide encapsulation (Table 1). The pI shifted to a more basic pH and the peptide’s net charge at the relevant pH values was, on average, incremented by 1.0 per additional lysine residue (Table 1 and Figure 1).

**Figure 2:**
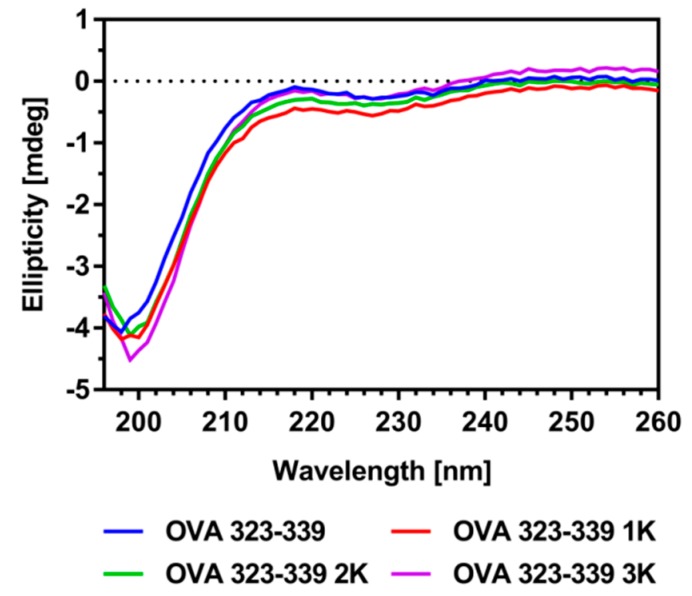
CD spectra of OVA 323-339 and its lysine-flanked variants. Representative blank-subtracted far-UV CD spectra are shown. Measurements were performed in triplicate in 5 mM PB sucrose pH 6.5 at a peptide concentration of 20 µM. CD spectra at other study-relevant conditions can be found in the supplement (Figure S1).

Taken together, these results suggest an increased impact of electrostatic interactions and hydrogen bonding on the overall interactions between cationic peptides and anionic lipid membranes at pH ≤ 6.5. Indications on the conservation of the peptide’s biological functionality (e.g., uptake, processing, presentation and recognition) were obtained from its secondary structure, which was determined by circular dichroism (CD) spectroscopy. The spectrum of OVA 323-339 shows the characteristics of a predominantly random coiled conformation which does not change upon the addition of flanked lysine residues (Figure 2). Moreover, measurements show that the conformation is independent of ionic strength and pH conditions relevant to its encapsulation into anionic liposomes (Figure S1).

### 3.2. Electrostatic Interactions of Peptides with Liposomes

**Figure 3:**
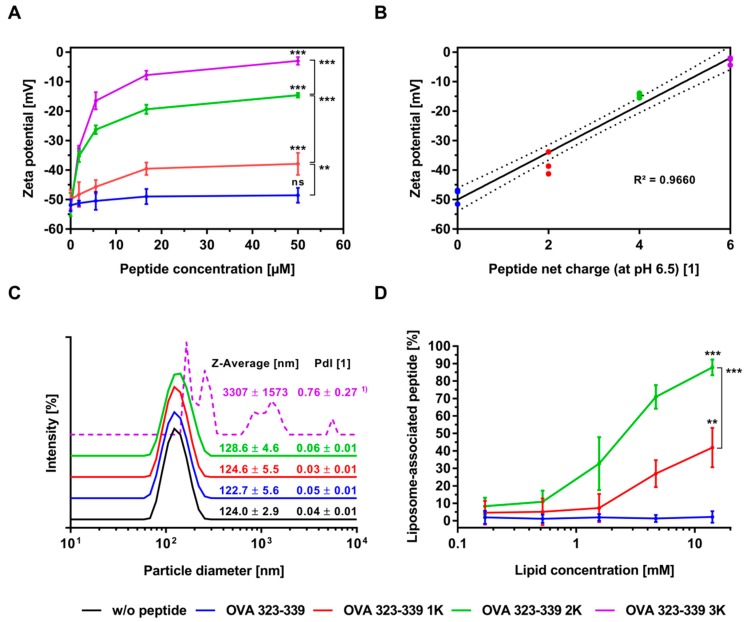
Electrostatic interactions of cationic peptides with anionic liposomes. (**A**) Zeta-potential measurements were performed at a constant lipid concentration of 200 µM and increasing concentrations of peptide in the range 0.62 µM to 50 µM. The corresponding molar peptide:lipid ratios are 1:324, 1:108, 1:36, 1:12 and 1:4. (**B**) Zeta potential (at a molar peptide:lipid ratio of 1:4) plotted against the peptide net charge of the respective peptide variant. Linear regression (best-fit line + 95% confidence bands) is shown. (**C**) Particle size analysis at a molar peptide:lipid ratio of 1:4. ^1)^ Particle size analysis did not fully comply with the requirements of the Zetasizer Nano ZS for a valid measurement (sample was too polydisperse). (**D**) Peptide binding studies were performed at a constant peptide concentration of 43 µM and increasing concentrations of lipid (liposomes) in the range 0.17 mM to 13.93 mM. The corresponding molar peptide:lipid ratios are 1:4, 1:12, 1:36, 1:108 and 1:324. Due to considerable blocking of the ultrafiltration membranes resulting from the loss of colloidal stability (i.e., agglomeration), OVA 323-339 3K was excluded from the binding studies. (**A**–**D**) All experiments were performed with anionic liposomes at a pH of 6.5, under low-ionic-strength conditions (0 mM NaCl). Data represents the mean ± standard deviation from three to nine experiments. Each data point of these three to nine experiments represents the average of three analytical replicates. Unless otherwise indicated, comparisons were always made with the OVA 323-339 experiment at the corresponding molar peptide:lipid ratio. Statistically significant differences are indicated by asterisks: *** *P* ≤ 0.001, ** *P* ≤ 0.01 and ns (not significant) *P* > 0.05, using a one-way ANOVA. A correction for multiple comparisons was made using Tukey’s test.

Zeta-potential studies and particle size analyses were performed to investigate the effects of lysine flanking on the electrostatic interactions between peptides and preformed liposomes. To systematically address the question of what controls these encapsulation-relevant interactions, in situ binding experiments were performed with liposomes of different charge under two ionic-strength conditions. Under low-ionic-strength conditions and a pH of 6.5, the addition of peptide caused a concentration-dependent reduction in the magnitude of the negative zeta potential of the anionic liposomes, whereas no reduction or only a minor reduction was observed at high ionic strength (Figure 3A and Table S3). The extent of this charge reduction was statistically significantly dependent on the net charge (i.e., the number of flanking lysine residues) of the peptide (Figure 3A,B). Interestingly, the results of the experiments with neutrally charged liposomes under low-ionic-strength conditions suggest a similar trend, although not as pronounced (Table S3).

These interactions are explained by the fact that the neutral liposome formulation (60 mol% DSPC/40 mol% cholesterol) used in this set of experiments exhibits a slightly negative zeta potential of −9.3 ± 2.4. All in all, these observations are in excellent agreement with published experimental data and theoretical considerations on the binding of charged peptides to liposomes of opposite charge [16,17].

Particle size analysis showed that under low-ionic-strength conditions the Z-Average of anionic liposomal suspensions is dramatically increased upon the addition of OVA 323-339 3K, while it remains unaffected upon the addition of the other peptide variants (Figure 3C). Additional experiments confirmed that this increase in Z-Average is a function of the ionic strength (Figure S2). The investigated molar peptide:lipid ratios of 1:324, 1:108, 1:36, 1:12 and 1:4 correspond to molar charge ratios of 1:8.1; 1:2.7; 1:0.9; 1:0.3 and 1:0.1, respectively. Under low-ionic-strength conditions, an increase in Z-Average was only observed at a molar charge ratio of 1:0.9 (i.e., Z = 1.1) and above (data not shown). The apparent particle size of neutral liposomes and liposomes prepared in a high-ionic-strength buffer was not affected by the addition of any of the peptide variants (Figure 3C and Table S4). Moreover, CD spectroscopy of the model peptides in the presence of liposomes revealed no conformational transitions (data not shown). In contrast to what has been reported in publications on the complexation of anionic liposomes with high molecular weight poly L-lysine, no restoration of the initial liposome diameter or charge conversion was observed when OVA 323-339 3K was added in excess over the accessible DSPG in the outer leaflet of the liposome, i.e., at molar charge ratios of 0.5 and above (Figure 3A,C) [40,41]. As poly L-lysine exhibits a considerably higher charge density as the investigated lysine-flanked peptide the agglomeration induced by these two distinct entities is likely to be different in nature [42]. Whatever the exact mechanism of agglomeration may be, our data unequivocally show that it is driven by electrostatic forces.

The conclusions drawn on the basis of the zeta-potential studies are confirmed by the binding studies shown in Figure 3D and Table S5. Lipid concentration-dependent binding to anionic liposomes at low-ionic-strength conditions was enabled and was statistically significantly increased by the addition of lysine residues to the N- and C-termini of the model peptide (Figure 3D). Control experiments with neutrally charged liposomes and liposomes prepared in a high-ionic-strength buffer showed no binding, or only minor binding, to the tested peptide variants (Table S5). More precisely, our experiments show that the addition of a charged membrane component is essential to achieve the desired electrostatically driven binding and that the extent of this binding is a function of the peptide’s net charge, among other things.

Moreover, these experiments show that the selected high-ionic-strength conditions (150 mM NaCl) are sufficient to impede binding by means of electric-field screening. These results also suggest that membrane partitioning and non-electrostatic interactions (e.g., hydrophobic interactions) do not, or only barely, contribute to the observed binding of our highly hydrophilic model peptide. Taken together, these findings clearly indicate the binding of the model peptides to be predominantly driven by electrostatic attractions.

**Figure 4:**
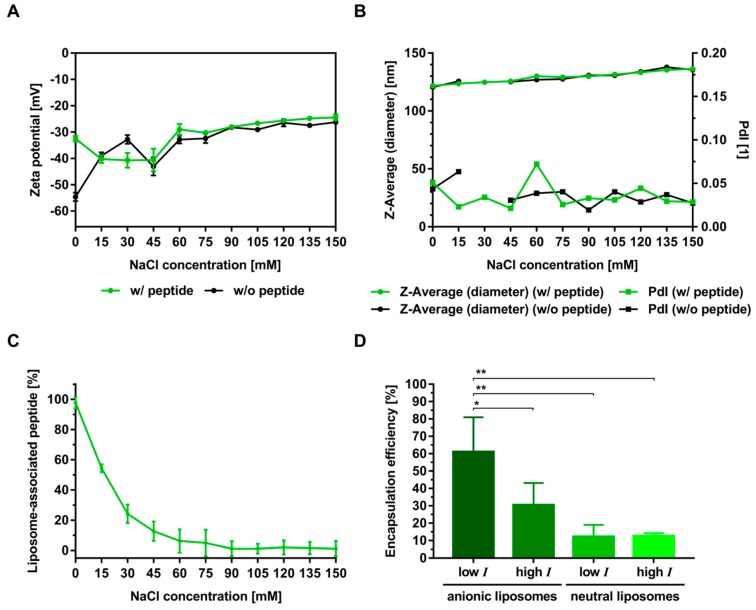
(**A**,**B**) Electrostatically driven binding and encapsulation of OVA 323-339 2K. (**A**) Zeta potential and (**B**) Z-Average/PdI of anionic liposomes in the presence (w/ peptide) and absence (w/o peptide) of OVA 323-339 2K. Data represent the mean ± standard deviation from three analytical replicates. Measurements with peptide were performed at a molar peptide:lipid ratio of 1:133 (3 µM peptide, 400 µM lipid). (**C**) Binding to anionic liposomes at a molar peptide:lipid ratio of 1:133 (50 µM peptide, 6.67 mM lipid). Unspecific binding to the ultrafiltration membrane at 0 mM NaCl and 150 mM NaCl was determined to be 13.1 ± 2.4% and 6.2 ± 2.8%, respectively. (**D**) Encapsulation efficiency at pH 6.5 and two different ionic-strength (I) conditions: low I (0 mM NaCl/300 mM sucrose) and high I (150 mM NaCl/0 mM sucrose). Both anionic and neutral liposomes were prepared by thin-film hydration and subsequent extrusion. The initial molar peptide:lipid ratio was 1:500 (10 µM peptide, 5 mM lipid). The results of the physico-chemical characterization of these peptide-loaded liposomes are summarized in the supplement (Table S6). (**A**–**C**) All experiments were performed with anionic liposomes in a 5 mM phosphate buffer with a constant osmolality and pH but with a varying ionic strength, i.e., varying concentrations of sodium chloride and sucrose. This was realized by mixing liposomes prepared in 5 mM PBS pH 6.5 (w/ 150 mM NaCl) with liposomes that were prepared in 5 mM PB sucrose pH 6.5 (w/ 300 mM sucrose). (**C**–**D**) Data represent the mean ± standard deviation from three experiments. Each data point of these three experiments represents the average of three analytical replicates. Statistically significant differences are indicated by asterisks: ** *P* ≤ 0.01 and * *P* ≤ 0.05, using a one-way ANOVA. A correction for multiple comparisons was made using Tukey’s test.

To further investigate the role of the ionic strength, OVA 323-339 2K was selected for additional interaction studies with anionic liposomes. Figure 4 shows results for the particle size, zeta potential and peptide binding as a function of the sodium chloride concentration of the buffer.

As expected, the magnitude of the negative zeta potential decreased with increasing ionic strength (Figure 4A). However, relevant differences in zeta potential between the experiments with peptide and those without were only observed at a sodium chloride concentration of 0 mM. The conductivity of the liposome suspension increased linearly and was not affected by the presence of a highly charged peptide variant (Figure S2B). These results clearly reflect the distinct contributions of charge screening by sodium chloride and binding of peptides, to the overall reduction in the magnitude of the negative zeta potential. Both particle diameter and PdI were not affected by the addition of peptide at any of the tested ionic strength conditions (Figure 4B). Binding decreased exponentially with increasing ionic strength (Figure 4C). In the absence of sodium chloride, > 98% of the peptide was bound to the anionic liposomes. The percentage of liposome-associated peptide at a sodium chloride concentration of around 15 mM was 50%, reaching its minimum (< 1%) at a concentration of 150 mM. Based on the results of these systematic screening experiments, encapsulation of hydrophilic peptides is expected to be most efficient at low-ionic-strength conditions. In contrast, the removal of non-encapsulated but electrostatically outer surface-associated peptide by methods based on ultrafiltration (e.g., tangential flow filtration, centrifugal ultrafiltration) or dialysis is expected to be most effective under high-ionic-strength conditions. Taken together, these experiments outline a straightforward strategy to control the electrostatic association/dissociation of hydrophilic peptides to charged lipid membranes.

### 3.3. Electrostatic-Driven Encapsulation of Peptides

Next, a series of experiments was performed to investigate whether the electrostatic binding observed in our exploratory in situ setup translates to corresponding levels of encapsulation efficiency. Two of the most widely used techniques in the lab-scale manufacturing of liposomes (thin-film hydration and microfluidic mixing) were employed to address this question.

To estimate the contribution of passive encapsulation, peptide-loaded liposomes were analyzed for particle size. In agreement with the findings of a recently published study on the encapsulation of a panel of physico-chemically diverse T-cell epitopes into cationic liposomes, physico-chemical characterization showed that the (type of) encapsulated peptide had no significant impact on parameters such as particle size and zeta potential [10]. Our analyses showed that the size of liposomes prepared by thin-film hydration was largely independent of parameters relevant to this study. Liposomes prepared by thin-film hydration had a median Z-Average diameter and PdI of 102 nm and 0.07, respectively (Table S6). In contrast, the size of liposomes prepared by microfluidic mixing showed a strong dependence on ionic strength and liposome charge (Table S7). While anionic liposomes prepared under low-ionic-strength conditions exhibited a Z-Average diameter around 60 nm, neutral liposomes or liposomes prepared under high-ionic-strength conditions had a Z-Average diameter of around 145 nm. The PdI of liposomes prepared by microfluidic mixing was in the range of 0.11 to 0.25. The observed differences in size can be explained by the fact that sucrose-containing buffers, such as those used for the preparation of liposomes under low-ionic-strength conditions, exhibit a higher dynamic viscosity. An increase in viscosity decreases the diffusion of coalescing lipid bilayer discs during the process of liposome formation, which eventually promotes the formation of smaller liposomes [43,44]. Moreover, repulsions between the negatively charged head groups of the phosphatidylglycerol soften the planar lipid bilayer discs, and thereby increase the vesiculation rate, which results in the formation of smaller liposomes [43,45]. Importantly, calculations indicate that particle size would only marginally contribute to the overall level of peptide encapsulation. The estimated fraction of captured volume for a liposome suspension at a total lipid concentration of 5 mM with a mean particle size of 60 nm and 145 nm, was estimated to be approximately 0.5% and 1.5% respectively [19].

**Figure 5:**
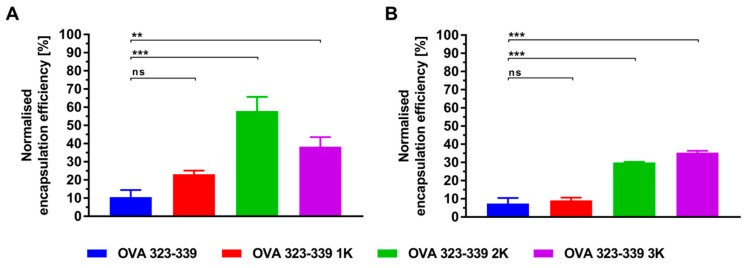
Electrostatically driven encapsulation of cationic peptides into anionic liposomes. Peptide-loaded liposomes were prepared by (**A**) thin-film hydration with subsequent downsizing by means of extrusion or (**B**) microfluidic mixing of an ethanolic lipid solution with an aqueous T helper cell peptide solution. Preparation took place at pH 6.5 under low-ionic-strength conditions (0 mM NaCl/300 mM sucrose). The initial molar peptide:lipid ratio was 1:667 (7.5 µM peptide, 5 mM lipid). The encapsulation efficiencies of the liposomes prepared by thin-film hydration were corrected for unspecific binding of non-encapsulated peptide to the membrane of the ultrafiltration unit. As part of that, the level of unspecific binding was normalized to that of OVA 323-339 (8.1 ± 1.9%). The calculated relative reduction in encapsulation efficiency was used to correct/normalize the encapsulation efficiencies of the peptide-loaded liposomes prepared on the microfluidics platform. The non-normalized/non-corrected encapsulation efficiencies are shown in the supplement (Figure S4). Data represent the mean ± standard deviation from three experiments. Statistically significant differences are indicated by asterisks: *** *P* ≤ 0.001, ** *P* ≤ 0.01 and ns (not significant), using a one-way ANOVA. A correction for multiple comparisons was made using Tukey’s test.

Bi-terminal sequence extension of the model peptide resulted in a statistically significantly increased encapsulation for both preparation techniques (Figure 5). Differences in the extent and particulars of this relation (i.e., the relation between encapsulation efficiency and the peptide’s net charge at a specific pH) between the two tested methods, suggest that additional factors other than electrostatic interactions (e.g., the underlying liposome formation mechanism, hydrophobic interactions, specific binding or spontaneous crowding) are involved in the encapsulation process. This conclusion is supported by recently published studies on the encapsulation of ovalbumin into neutral liposomes that report striking method-dependent differences in encapsulation efficiency [20].

While microfluidic mixing was reported to result in an ovalbumin encapsulation of around 35%, methods based on mechanical dispersion (i.e., sonication and extrusion) were reported to yield encapsulation efficiencies in the range 5% to 10%. Moreover, studies on the preparation of ovalbumin-loaded liposomes prepared by thin-film hydration (without extrusion) report an unexpectedly high and ionic-strength-independent encapsulation of ovalbumin into anionic liposomes, at around 30% [13]. Taken together, these reports indicate that hydrophobic interactions and/or specific lipid-binding properties of the protein are involved in the encapsulation process. Differences in encapsulation efficiency among different preparation techniques might therefore be explained by the circumstance that intramolecular hydrophobic interactions, and thus probably also hydrophobic protein-membrane interactions, are affected by the presence of organic solvents [46].

**Figure 6:**
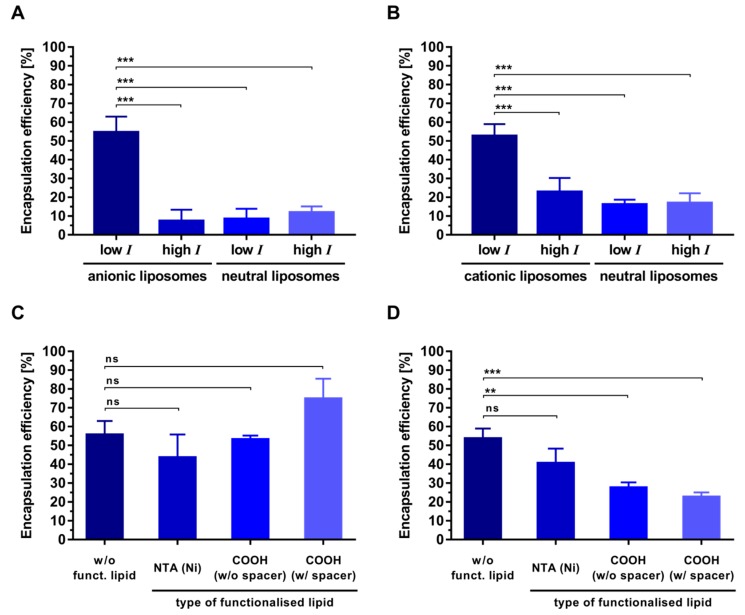
Electrostatically driven encapsulation of OVA 323-339 into liposomes of different composition. (**A**–**D**) Electrostatically driven encapsulation of OVA 323-339 into (**A**) anionic liposomes at pH 4.0, (**B**) cationic liposomes at pH 8.5, (**C**) functionalized anionic liposomes at pH 4.0, and (**D**) functionalized cationic liposomes at pH 8.5. (**A**,**B**) Encapsulation efficiency under different ionic-strength (I) conditions: low I (0 mM NaCl/300 mM sucrose) and high I (150 mM NaCl/0 mM sucrose). (**C**,**D**) All preparation took place under low-ionic-strength conditions. Liposomes were prepared by thin-film hydration with subsequent extrusion. The initial molar peptide:lipid ratio was 1:500 (10 µM peptide, 5 mM lipid). Data represent the mean ± standard deviation from three experiments. Statistically significant differences are indicated by asterisks: *** *P* ≤ 0.001, ** *P* ≤ 0.01 and ns (not significant), using a one-way ANOVA. A correction for multiple comparisons was made using Tukey’s test.

To investigate the role of pH, liposome composition and ionic strength in controlling peptide encapsulation, OVA 323-339 2K and OVA 323-339 were selected for additional experiments. The encapsulation of OVA 323-339 2K into anionic liposomes under low-ionic-strength conditions at pH 6.5 was significantly higher than that into neutral liposomes and that under high-ionic-strength conditions (Figure 4D). The efficiency with which OVA 323-339 was encapsulated into anionic liposomes at pH 4.0 was considerably higher than that with which it was encapsulated into charged liposomes at its calculated isoelectric point of 6.5 (Figure 5A and Figure 6A). The same is true for its encapsulation into cationic liposomes at pH 8.5 (Figure 5A and Figure 6B). Encapsulation of OVA 323-339 into charged liposomes under low-ionic-strength conditions was consistently higher than that into neutral liposomes and that under high-ionic-strength conditions (Figure 6A,B and Figure S3). The results of these encapsulation studies are largely in agreement with the results of our interaction studies and substantiate the suggested electrostatically driven encapsulation mechanism. It is worth noting that encapsulation experiments performed under conditions that favor high electrostatic interactions consistently resulted in encapsulation efficiencies of around 50% (Figure 4D and Figure 6A,B).

This finding is in excellent agreement with well-established models on the formation of unilamellar liposomes, which imply a maximum encapsulation efficiency of 50% in the case of an exclusively electrostatically driven entrapment process [47,48].

The experiments with OVA 323-339 at various pH values are consistent with predictions made based on the net charge/pH profile, which suggests that encapsulation at the isoelectric point occurs in a primarily (passive) non-electrostatic manner. While OVA 323-339 has an overall neutral charge at pH 6.5, it exhibits a net charge of +2.5 and −1.7 at pH 4.0 and pH 8.5, respectively (Figure 1). We therefore infer that the low encapsulation efficiencies (around 10%) are a direct consequence of low or absent peptide–membrane interactions during the liposome formation process. Data from recent reports on the simultaneous encapsulation of OVA 323-339 and SLP OVA24 (an extended variant of OVA 257-264 with a pI of 4.2) into cationic liposomes at pH 8.5, support these conclusions [49]. At pH 8.5, OVA 323-339 exhibits a net charge of −1.7, while that of SLP OVA24 is around −3.8. The reported encapsulation efficiencies were around 20% and 46%, respectively. In agreement with our data, these findings support the idea that encapsulation efficiency is, inter alia, a function of the peptide’s net charge/pH profile and the liposome’s electric charge. In contrast, data from recently published work on the encapsulation of a panel of 15 physico-chemically diverse NBD-labelled peptides into cationic liposomes indicate encapsulation efficiency to be hardly predictable [10]. Neither the peptide’s net charge nor its hydropathicity seem to correlate with the extent of encapsulation. This contrary finding might be explained by the marked differences in aqueous solubility of the employed peptides as well as by the considerably different production, purification, sample preparation and quantitative analysis of the peptide-loaded liposomes. Taken together, these results have interesting implications regarding the electrostatically driven encapsulation of peptides with unfavorable physico-chemical properties and the ratio-controlled encapsulation of multiple peptides. It is, for example, well established that preparation and lengthy processing of liposomes at highly acidic or basic pH values, promote the degradation of phospholipids to fatty acids and lysophosholipids [50]. Figure 5 shows that shifting the peptide’s pI by adding charged amino acid residues to its N- and C-termini is a viable approach to enabling and enhancing the encapsulation at more favorable pH values. Moreover, N- and C-terminal sequence extension can be thought of as a strategy for improving the solubility of hydrophobic peptides [51].

As this study was carried out within the framework of our ongoing efforts to develop T helper liposomes (i.e., liposomes that carry T helper cell peptides within their interior and display immunogens on their surface) we selected three types of functionalized lipids in order to investigate their effect on the electrostatically driven encapsulation of our model peptide into functionalized liposomes of relevant membrane composition. The addition of 4 mol% NTA (Ni)-functionalized lipid did not affect the encapsulation efficiency of OVA 323-339 (Figure 6C,D). Likewise, the addition of 4 mol% carboxyl-functionalized lipid did not impair the encapsulation into anionic liposomes (Figure 6C). In contrast, it reduced peptide encapsulation into cationic liposomes in a statistically significant manner (Figure 6D). These observations are in part explained by the fact that the carboxyl-functionalized lipids are negatively charged. In concordance with that, the zeta potential of cationic liposomes was considerably reduced by the addition of carboxyl-functionalized lipids, while that of anionic liposomes was not affected at all (Table S6). Bi-terminal sequence extension of rather basic/neutral peptides such as OVA 323-339, with negatively charged amino acids (e.g., aspartic acid or glutamic acid) could be a viable approach to counter-act these losses in encapsulation efficiency. This approach is of particular interest when encapsulating peptides under basic conditions (in order to increase the magnitude of the peptides’s negative net charge) is not advised, or when changing the membrane composition is not compatible with the vaccine’s anticipated mode of action or subsequent processing steps. For instance, covalent, epitope-preserving conjugation of tag-free, anionic HIV-1 envelope trimers onto the surface of peptide-loaded liposomes necessitates a positive membrane charge in order to establish the levels of preconcentration required for the conjugation reaction to proceed with high efficiency (Suleiman et al., manuscript in preparation).

Altogether, using the example of a widely employed model peptide, these experiments show that the studied electrostatic-based approach can be applied to efficiently encapsulate hydrophilic peptides into a range of functionalized liposomes that could potentially be used in the preparation of protein-liposome conjugates such as T helper liposomes.

### 3.4. In Vitro Co-Cultivation

CD spectroscopy gave first indications regarding the preservation of the peptide’s biological function upon the bi-terminal addition of lysine residues (Figure 2). A co-culture of dendritic cells and antigen-specific CD4^+^ T cells was set up to further investigate the effect of this sequence extension on the recognition of MHC-II/peptide complexes by T-cell receptors (TCRs) of relevant immune cells. We co-cultured splenic DCs with naïve antigen-specific CD4^+^ T cells from OT-II TCR transgenic mice, which proliferate upon recognition of the MHC-II/OVA 323-339 complexes [52].

To allow a conclusive interpretation of these experiments free (non-liposomal) peptides and liposomes encapsulating peptide were subjected to a stability study under cell culture conditions (w/o cells). The concentration of free OVA 323-339 exponentially decreased to a non-detectable level over a time frame of 72 h (Figure S5A). Both OVA 323-339 1K and OVA 323-339 2K exhibit similar stability profiles, in which around 50% of the initial peptide content could be detected after 72 h of incubation. Interestingly, in case of OVA 323-339 3K, around 90% could be recovered. Elucidation of the mechanisms that led to this differential peptide degradation was beyond the scope of this study. Peptides encapsulated in liposomes exhibit a distinct stability profile in which 90% to 100% of the initial peptide content could be recovered after 72 h (Figure S5B). Taken together, the results indicate that the peptides remain stably encapsulated under the cell culture conditions relevant to our co-cultivation experiments.

**Figure 7:**
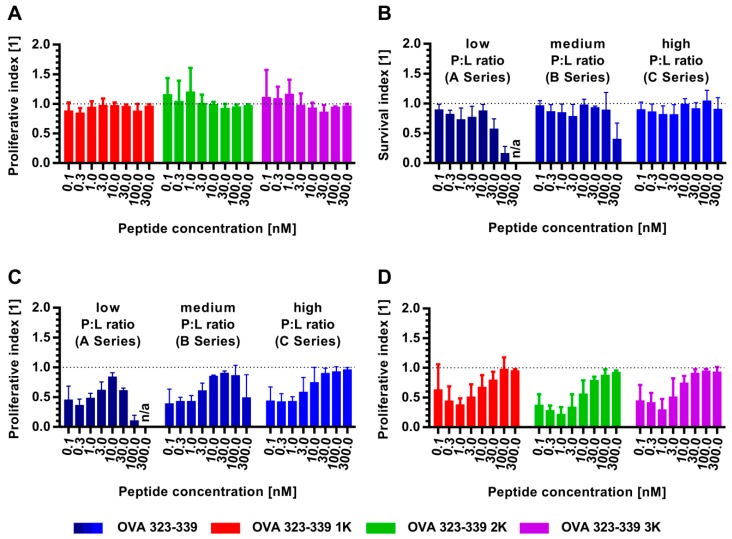
Co-culture of dendritic cells and antigen-specific CD4^+^ T cells in the presence of free (non-liposomal) or liposomal peptide. (**A**) Influence of bi-terminal sequence extension on the proliferation of antigen-specific CD4^+^ T cells. The results are presented as a proliferative index (% of proliferating CD4^+^ T cells cultivated with the bi-terminally extended peptide / % of proliferating CD4^+^ T cells cultivated with the unmodified peptide). (**B**) Influence of the peptide:lipid ratio of peptide-loaded liposomes on the viability of the antigen-specific CD4^+^ T cells. The results are presented as a survival index (% of viable CD4^+^ T cells cultivated with liposomal OVA 323-339 / % of viable CD4^+^ T cells cultivated with free OVA 323-339). Viability was determined by flow cytometry after staining with Fixable Viability Dye from Thermo Fisher Scientific (Figure S6). (**C**) Influence of the peptide:lipid ratio on the proliferation of antigen-specific CD4^+^ T cells. The results are presented as a proliferative index (% of proliferating CD4^+^ T cells cultivated with liposomal OVA 323-339 / % of proliferating CD4^+^ T cells cultivated with free OVA 323-339). (**D**) Differences in proliferation of antigen-specific CD4^+^ T cells in response to liposomal and free peptides. Peptide-loaded liposomes of the C series were used. The results are presented as a proliferative index (% of proliferating CD4^+^ T cells cultivated with liposomal peptide / % of proliferating CD4^+^ T cells cultivated with free peptide). (**B-D**) The peptide-loaded liposomes used in this set of in vitro experiments were composed of 45 mol% DSPC, 40 mol% cholesterol and 15 mol% DSPG. A comprehensive characterization of these liposomes is summarized in Table S8 and Table S9. (**A–D**) Data represent the mean ± standard deviation from three experiments.

Co-cultivation experiments showed that OVA 323-339 and all of its lysine-flanked variants induced a dose-dependent proliferation (Figure S7). At low (suboptimal) peptide concentrations, OVA 323-339 2K and OVA 323-339 3K appear to induce a slightly higher proliferation than the unmodified peptide (Figure 7A and Figure S7).

This might be a consequence of the increased stability/availability of these peptide variants in the culture medium (Figure S5A). In excellent agreement with the CD spectroscopy data, our experiments demonstrate that the bi-terminal sequence extension of OVA 323-339 does not affect the TCR-mediated proliferation of T helper cells in a negative manner (Figure 7A and Figure S7).

Next, we investigated the impact of varying molar peptide:lipid ratios on the kinetics of T-cell proliferation in vitro. All liposomal peptide formulations used in this in vitro study were prepared using a thin-film hydration protocol. Non-encapsulated material was removed by tangential flow filtration run under high-ionic-strength conditions. The peptide-loaded liposomes showed a highly negative zeta potential of around −58 mV and a mean Z-Average diameter and PdI of 125 nm and 0.05, respectively (Table S8). Preparations at various molar peptide:lipid ratios allowed the formation of liposomes with an average number of peptides in the order of magnitude of 10, 100 or 1000 per liposome (Table S9). The encapsulation efficiency was in the range 30% to 55% (Table S9). Furthermore, the data indicate improved encapsulation efficiency with increasing numbers of flanked lysine residues, which is in excellent agreement with the conclusions drawn on the basis of our extensive cross-platform encapsulation studies shown in Figure 5. The viability of the CD4^+^ T cells in co-culture was reduced as function of the peptide:lipid ratio of the used peptide-loaded liposomes (Figure 7B). In agreement with our expectations, liposomes with a low peptide:lipid ratio (A series: ~10 peptide molecules per liposome) caused a higher reduction in viability than those with a medium peptide:lipid ratio (B series: ~100 peptide molecules per liposome). The reduction in viability at high peptide concentrations observed for these liposome formulations is a consequence of the high lipid concentrations and is probably the reason for the reduced proliferation of the T cells in co-culture (Figure 7C and Figure S8). Liposomes of the C series (~1000 peptide molecules per liposome) have a more favorable peptide:lipid ratio and were shown to have no negative impact on T-cell viability over the tested peptide concentration range of 0.1 nM to 300 nM. The corresponding total lipid concentration at a particular peptide concentration can be derived from the data shown in Table S9. In the case of the peptide-loaded liposomes of the C series, a peptide concentration of 300 nM corresponds to a total lipid concentration of approximately 40 µM. Lipid concentrations higher than 250 µM were found to be critical with regard to the viability of T cells in culture (data not shown). Therefore, liposomes with a high peptide:lipid ratio were selected as the preferred peptide delivery vehicle for subsequent experiments in which the T-cell proliferation in response to different liposomal peptide variants was tested. Although the T-cell viability in response to the addition of liposomal peptide was comparable to that after the addition of free peptide, liposomal peptides induced lower T-cell proliferation (Figure 7C,D). Interestingly, this effect was peptide concentration-dependent, being more pronounced at lower peptide concentrations. This observation was confirmed for all peptide variants in the context of the C series and might be a consequence of different mechanisms involved in the MHC-II loading of liposomal and free peptides. Altogether, these experiments show that bi-terminal addition of charged amino acids does not affect the hydrophilic model peptide’s ability to induce proliferation of antigen-specific T helper cells.

## 4. Conclusions

The controlled and efficient encapsulation of peptides and proteins into functionalized liposomes is of high relevance to a multitude of scientific fields. The present study systematically addressed the question of what controls the electrostatically driven encapsulation of hydrophilic peptides into liposomes. In agreement with published experimental work and theoretical considerations, we have identified ionic strength, pH, liposome charge and the peptide’s net charge/pH profile as crucial parameters controlling the electrostatically driven encapsulation of peptides. We were able to show that the employed methods of studying the interactions between peptides and liposomes are of high value in the search for optimal manufacturing and processing conditions. The findings of our extensive screening studies were confirmed in encapsulation experiments in which we used two widely employed methods for the preparation of liposomes. Notably, the findings reported herein also have important implications for the ionic strength-dependent removal of non-encapsulated, liposome-associated peptides by ultrafiltration-based methods such as tangential flow filtration. Moreover, we were able to demonstrate that the bi-terminal sequence extension of hydrophilic non-conformational peptide epitopes offers a promising approach for enabling and improving their electrostatically driven encapsulation into liposomes. Extensive characterization of the bi-terminally flanked model peptide and a set of in vitro experiments suggest that this approach may also be applicable to other hydrophilic T helper cell epitopes. The strategy presented here provides a basis for the scalable, efficient, electrostatically driven encapsulation of peptides in the context of the manufacturing of T helper liposomes—a modular liposome-based vaccine that aims at the recruitment of heterologous T helper cells in order to improve and modulate immune responses towards liposome-displayed immunogens. As many biologically relevant peptide epitopes are considerably less hydrophilic than the model peptides employed in this study, future investigations will attempt to apply this approach to a set of hydrophobic T helper cell peptides. Furthermore, upcoming studies will explore the bi-terminal addition of negatively charged amino acids as a strategy to modulate the electrostatically driven encapsulation of peptides into functionalized cationic liposomes.

## Figures and Tables

**Table 1:** Sequences and physico-chemical properties of the model T helper cell peptides.

Peptide Variant	Sequence	GRAVY [1] ^1^	Isoelectric Point [1] ^2^
OVA 323-339	ISQAVHAAHAEINEAGR	−0.299	6.5
OVA 323-339 1K	KISQAVHAAHAEINEAGRK	−0.616	8.9
OVA 323-339 2K	KKISQAVHAAHAEINEAGRKK	−0.929	10.0
OVA 323-339 3K	KKKISQAVHAAHAEINEAGRKKK	−1.187	10.3

^1^ Calculated using ProtParam [36]. ^2^ Calculated using PROTEIN CALCULATOR v3.4 [37].

## Supplementary Materials

The following are available online at https://www.mdpi.com/1999-4923/11/11/619/s1, Figure S1: CD spectra of the model T helper cell peptide and its variants, Figure S2: Electrostatic interactions of OVA 323-339 3K with anionic liposomes, Figure S3: Effect of liposome composition and ionic strength on the encapsulation efficiency of OVA 323-339, Figure S4: Encapsulation of the model T helper cell peptides into anionic liposomes (non-normalized/non-corrected), Figure S5: Stability of free and liposomal model T helper cell peptide under cell culture conditions, Figure S6: Gating strategy for the experiments shown in Figure 7, Figure S7: Antigen-specific CD4^+^ T-cell proliferation induced by various free (non-liposomal) OVA 323-339 variants, Figure S8: Antigen-specific CD4^+^ T-cell proliferation induced by liposomal OVA 323-339, Table S1: Calibration and validation of the peptide HPLC method, Table S2: Calibration and validation of the lipid HPLC method, Table S3: Zeta potential of liposomes upon the addition of peptides (control experiments), Table S4: Z-Average particle size and PdI of liposomes upon the addition of peptides (control experiments), Table S5: Binding of the model T helper cell peptide and its variants to liposomes (control experiments), Table S6: Physico-chemical characterization of peptide-loaded liposomes prepared by thin-film hydration, Table S7: Physico-chemical characterization of peptide-loaded liposomes prepared by microfluidic mixing, Table S8: Physico-chemical characterization of peptide-loaded anionic liposomes used in the in vitro co-cultivation, Table S9: Lipid recovery, peptide content and encapsulation efficiency of anionic liposomes used in the in vitro co-cultivation.
