# Supplementary material for: Electrostatically Driven Encapsulation of Hydrophilic, Non-Conformational Peptide Epitopes into Liposomes
Source: Pharmaceutics. 2019 Nov 18;11(11):619. doi: 10.3390/pharmaceutics11110619 (PMC6920922; doi:10.3390/pharmaceutics11110619)
Supplement: Supplementary file 1 [file pharmaceutics-11-00619-s001.pdf]

pharmaceutics

Supplementary Materials: Electrostatically Driven Encapsulation of Hydrophilic, Non-Conformational Peptide Epitopes into Liposomes

Ehsan Suleiman, Dominik Damm, Mirjam Batzoni, Vladimir Temchura, Andreas Wagner, Klaus Überla and Karola Vorauer-Uhl

Table S1. Calibration and validation of the peptide HPLC method.

Peptide variant	Calibration range [µM]	LOD [µM]	LOQ [µM]	Retention time [min]
**OVA 323-339**	0.33 - 33.42	0.03	0.08	9.4
**OVA 323-339 1K**	0.83 - 29.64	0.21	0.63	10.0
**OVA 323-339 2K**	0.93 - 25.95	0.24	0.72	9.8
**OVA 323-339 3K**	1.16 - 26.09	0.14	0.43	9.7

Note: The R^2 values of the linear calibration curves were in the range 0.9986 to 0.9995. Residual plots indicated the appropriateness of the linear model. LOD and LOQ were calculated on the basis of the standard deviation of the y-intercept, according to the ICH guideline Q2 (R1): "Validation of Analytical Procedures: Text and Methodology (2005)". The acceptable range for control standard recovery was set to 85-115 %.

Table S2. Calibration and validation of the lipid HPLC method.

Lipid	Calibration range [mM]	LOD [mM]	LOQ [mM]	Retention time [min]	Gain setting
**Cholesterol**	0.16 - 0.65	0.07	0.11	20.4	5
**DSPC**	0.18 - 0.96	0.06	0.09	21.6	5
**DSPG**	0.03 - 0.24	0.02	0.02	22.9	5

Note: The R^2 values of the quadratic calibration curves were in the range 0.9987 to 0.9996. Residual plots indicated the appropriateness of the quadratic model. LOD and LOQ were estimated on the basis of the standard deviation of the lowest calibration point, according to the ICH guideline Q2 (R1): "Validation of Analytical Procedures: Text and Methodology (2005)". The acceptable range for control standard recovery was set to 85-115 %.

Table S3. Zeta potential of liposomes upon the addition of peptides (control experiments).

Zeta potential [mV]		w/o peptide (n=9)	Peptide variant (n=3)			
			OVA 323-339	OVA 323-339 1K	OVA 323-339 2K	OVA 323-339 3K
Buffer / liposome formulation	low ionic strength / neutral liposomes	-9.3 ± 2.4	-7.6 ± 1.5	-6.9 ± 2.6	-3.3 ± 2.7	-2.6 ± 0.5
	high ionic strength / anionic liposomes	-23.4 ± 1.4	-23.5 ± 0.3	-18.0 ± 5.1	-22.7 ± 0.8	-21.2 ± 2.4
	high ionic strength / neutral liposomes	-4.4 ± 0.7	-4.1 ± 0.9	-3.2 ± 0.3	-3.2 ± 1.3	-1.7 ± 2.6

Note: Experiments were performed at a molar peptide:lipid ratio of 1:4 (50 µM peptide, 200 µM lipid). Data represent the mean ± standard deviation from three to nine experiments. Each data point of these three to nine experiments represents the average of three analytical replicates.

Table S4. Z-Average particle size and PdI of liposomes upon the addition of peptides (control experiments).

Z-Average (diameter) [nm] / PdI [1]		w/o peptide (n=9)	Peptide variant (n=3)			
			OVA 323-339	OVA 323-339 1K	OVA 323-339 2K	OVA 323-339 3K
Buffer / liposome formulation	low ionic strength / neutral liposomes	126.4 ± 2.8	124.8 ± 5.6	124.2 ± 4.8	124.4 ± 6.4	124.7 ± 5.9
		0.04 ± 0.01	0.04 ± 0.01	0.05 ± 0.01	0.04 ± 0.01	0.04 ± 0.01
	high ionic strength / anionic liposomes	131.3 ± 2.5	130.4 ± 4.1	130.2 ± 4.7	129.7 ± 4.8	131.0 ± 4.0
		0.05 ± 0.01	0.04 ± 0.01	0.05 ± 0.02	0.04 ± 0.00	0.04 ± 0.01
	high ionic strength / neutral liposomes	134.0 ± 2.8	131.8 ± 4.5	132.1 ± 5.0	131.7 ± 4.9	132.5 ± 4.5
		0.08 ± 0.03	0.06 ± 0.01	0.08 ± 0.01	0.07 ± 0.00	0.06 ± 0.01

Note: Experiments were performed at a molar peptide:lipid ratio of 1:4 (50 µM peptide, 200 µM lipid). Data represent the mean ± standard deviation from three to nine experiments. Each data point of these three to nine experiments represents the average of three analytical replicates.

Table S5. Binding of the model T helper cell peptide and its variants to liposomes (control experiments).

<table>
<tr><th colspan="2" rowspan="2">Fraction of bound peptide [%]</th><th colspan="3">Peptide variant</th></tr>
<tr><th>OVA 323-339</th><th>OVA 323-339 1K</th><th>OVA 323-339 2K</th></tr>
<tr><td rowspan="3">Buffer / liposome formulation</td><td>low ionic strength / neutral liposomes</td><td>-0.8 ± 6.4</td><td>3.5 ± 2.5</td><td>3.8 ± 7.3</td></tr>
<tr><td>high ionic strength / anionic liposomes</td><td>4.6 ± 6.5</td><td>6.9 ± 3.7</td><td>8.8 ± 2.4</td></tr>
<tr><td>high ionic strength / neutral liposomes</td><td>6.0 ± 5.7</td><td>9.1 ± 5.4</td><td>10.6 ± 3.0</td></tr>
</table>

Note: Experiments were performed at a molar peptide:lipid ratio of 1:324 (43 µM peptide, 14.08 mM lipid). Data represent mean ± standard deviation from three experiments.

Table S6. Physico-chemical characterization of peptide-loaded liposomes prepared by thin-film hydration.

Ionic strength	pH [1]	Lipid formulation	Peptide variant	Z-Average [nm]	PdI [1]	Zeta potential [mV]
Low	4.0	anionic	OVA 323-339	102.6 ± 1.0	0.07 ± 0.01	-39.4 ± 0.7
Low	4.0	neutral	OVA 323-339	110.4 ± 5.1	0.07 ± 0.01	-7.1 ± 0.5
High	4.0	anionic	OVA 323-339	100.9 ± 1.1	0.06 ± 0.00	-17.3 ± 0.8
High	4.0	neutral	OVA 323-339	104.2 ± 1.7	0.05 ± 0.02	-1.1 ± 0.6
Low	4.0	anionic (w/ 16:0 Dodecanoyl PE)	OVA 323-339	90.0 ± 4.9	0.09 ± 0.01	-42.1 ± 0.7
Low	4.0	anionic (w/ DSPE-PEG14-COOH)	OVA 323-339	97.6 ± 1.5	0.07 ± 0.01	-40.0 ± 0.5
Low	4.0	anionic (w/ 18:1 DGS-NTA(Ni))	OVA 323-339	99.8 ± 1.1	0.08 ± 0.01	-42.9 ± 1.4
Low	8.5	cationic	OVA 323-339	89.2 ± 2.1	0.10 ± 0.00	39.5 ± 3.6
Low	8.5	neutral	OVA 323-339	96.0 ± 1.0	0.08 ± 0.02	-14.9 ± 4.1
High	8.5	cationic	OVA 323-339	100.9 ± 2.8	0.07 ± 0.02	12.0 ± 0.9
High	8.5	neutral	OVA 323-339	104.0 ± 0.4	0.06 ± 0.02	-1.7 ± 2.0
Low	8.5	cationic (w/ 16:0 Dodecanoyl PE)	OVA 323-339	105.3 ± 1.5	0.12 ± 0.01	23.5 ± 3.9
Low	8.5	cationic (w/ DSPE-PEG14-COOH)	OVA 323-339	101.2 ± 2.1	0.10 ± 0.02	29.6 ± 0.2
Low	8.5	cationic (w/ 18:1 DGS-NTA(Ni))	OVA 323-339	99.8 ± 2.2	0.09 ± 0.01	36.6 ± 2.1
Low	6.5	anionic	OVA 323-339	103.9 ± 2.7	0.06 ± 0.00	-51.4 ± 0.6
Low	6.5	anionic	OVA 323-339 1K	101.8 ± 1.3	0.07 ± 0.01	-52.6 ± 1.7
Low	6.5	anionic	OVA 323-339 2K	102.2 ± 1.5	0.07 ± 0.01	-49.1 ± 1.0
Low	6.5	neutral	OVA 323-339 2K	114.3 ± 1.8	0.07 ± 0.01	-8.9 ± 0.4
High	6.5	anionic	OVA 323-339 2K	115.1 ± 4.2	0.07 ± 0.01	-17.9 ± 1.1
High	6.5	neutral	OVA 323-339 2K	121.1 ± 0.3	0.06 ± 0.02	-2.6 ± 1.3
Low	6.5	anionic	OVA 323-339 3K	107.5 ± 8.5	0.11 ± 0.07	-50.5 ± 0.3

Note: Data represent mean ± standard deviation from two to three experiments. Each data point of these two to three experiments represents the average of three analytical replicates.

Table S7. Physico-chemical characterization of peptide-loaded liposomes prepared by microfluidic mixing.

Ionic strength	pH [1]	Lipid formulation	Peptide variant	Z-Average [nm]	PdI [1]
Low	6.5	anionic	OVA 323-339	59.5 ± 0.3	0.17 ± 0.01
Low	6.5	anionic	OVA 323-339 1K	59.8 ± 0.8	0.17 ± 0.01
Low	6.5	anionic	OVA 323-339 2K	59.7 ± 0.2	0.17 ± 0.00
Low	6.5	anionic	OVA 323-339 3K	61.0 ± 0.8	0.16 ± 0.01
Low	4.0	anionic	OVA 323-339	49.2 ± 1.0	0.25 ± 0.00
High	4.0	anionic	OVA 323-339	153.2 ± 3.0	0.21 ± 0.01
Low	4.0	neutral	OVA 323-339	139.2 ± 1.5	0.12 ± 0.02
High	4.0	neutral	OVA 323-339	138.9 ± 0.9	0.11 ± 0.04

Note: Data represent mean ± standard deviation from two to three experiments.

Table S8. Physico-chemical characterization of peptide-loaded anionic liposomes used in the in vitro co-cultivation.

Sample code	Peptide variant	Z-Average [nm]	PdI [1]	Zeta potential [mV]
0K/A	OVA 323-339	126.3 ± 0.7	0.10 ± 0.02	-59.5 ± 0.9
0K/B		126.6 ± 0.1	0.05 ± 0.00	-59.5 ± 0.7
0K/C		124.7 ± 0.6	0.04 ± 0.01	-60.5 ± 0.5
0K/X		126.0 ± 0.3	0.05 ± 0.01	-58.6 ± 3.0
0K/Y		126.2 ± 0.8	0.05 ± 0.01	-58.3 ± 0.9
0K/Z		124.9 ± 1.5	0.05 ± 0.02	-55.7 ± 2.5
1K/A	OVA 323-339 1K	125.5 ± 1.3	0.05 ± 0.01	-54.8 ± 3.0
1K/B		125.0 ± 0.7	0.05 ± 0.00	-57.4 ± 1.2
1K/C		146.7 ± 1.4	0.07 ± 0.01	-59.1 ± 0.8
2K/A	OVA 323-339 2K	121.4 ± 0.9	0.04 ± 0.01	-59.2 ± 1.1
2K/B		120.0 ± 0.5	0.04 ± 0.01	-58.0 ± 0.9
2K/C		124.1 ± 0.9	0.06 ± 0.02	-57.3 ± 0.6
3K/A	OVA 323-339 3K	126.2 ± 0.1	0.05 ± 0.02	-57.5 ± 1.0
3K/B		123.4 ± 2.3	0.04 ± 0.03	-58.0 ± 0.5
3K/C		123.0 ± 0.6	0.05 ± 0.03	-57.2 ± 0.9

Note: Peptide-loaded anionic liposomes were prepared with an initial molar peptide:lipid ratio of 1:6667 (A and X series), 1:667 (B and X series) or 1:67 (C and Z series). All lipid films were rehydrated with 5 mM PB sucrose pH 6.5, except for those of the 0K/A, 0K/B and 0K/C series, which were rehydrated with 5 mM AB sucrose pH 4.0. Data represent the mean of three analytical replicates ± standard deviation from single liposome batches after removal of non-encapsulated peptide.

Table S9. Lipid recovery, peptide content and encapsulation efficiency of anionic liposomes used in the in vitro co-cultivation.

Sample code	Peptide variant	Lipid recovery [%]	Peptide concentration [µM]	Peptides per liposome (average) [1]	Encapsulation efficiency [%]
0K/A	OVA 323-339	82.4	0.8 ± 0.0	9	49.1 ± 0.9
0K/B		81.6	8.5 ± 0.2	113	54.5 ± 1.1
0K/C		88.2	73.9 ± 0.7	949	47.5 ± 0.5
0K/X		68.9	- 1)	- 1)	- 1)
0K/Y		68.6	0.3 ± 0.0	4	1.9 ± 0.1
0K/Z		74.8	4.2 ± 0.1	54	2.6 ± 0.0
1K/A	OVA 323-339 1K	96.1	0.4 ± 0.0	6	27.5 ± 2.7
1K/B		100.8	5.3 ± 0.1	70	34.6 ± 0.5
1K/C		86.9	47.3 ± 0.7	821	30.6 ± 0.4
2K/A	OVA 323-339 2K	70.9	0.6 ± 0.0	7	36.4 ± 1.1
2K/B		76.4	6.1 ± 0.1	72	38.3 ± 0.6
2K/C		63.9	56.6 ± 1.6	691	35.6 ± 1.0
3K/A	OVA 323-339 3K	101.3	0.5 ± 0.1	7	36.3 ± 3.4
3K/B		89.0	6.6 ± 0.3	83	43.6 ± 1.7
3K/C		78.8	66.8 ± 2.4	830	44.4 ± 1.6

Note: Peptide concentrations were normalized to a total lipid concentration of 10 mM. The average number of peptides per liposome was calculated on the basis of the particle size distribution by number, the lipid concentration and the peptide concentration of the purified liposomes. Unilamellarity and sphericity of the liposomes as well as an average bilayer thickness of 5 nm and head group area of 0.5 nm² were assumed in performing these estimations. The encapsulation efficiency was calculated on the basis of the nominal peptide concentration after hydration of the lipid film and that after purification by means of TFF. Calculations of the encapsulation efficiency were performed taking into account the preparation-related lipid losses. Lipid recovery was calculated on the basis of the nominal lipid concentration at the level of film-hydration and the lipid concentration of the purified liposomes. Data represent the mean ± standard deviation of two (lipid quantification) or three (peptide quantification) analytical replicates of a single liposome preparation.

1) Peptide was not detectable. Therefore, it was not possible to estimate the average number of peptides per liposome or to determine the encapsulation efficiency.

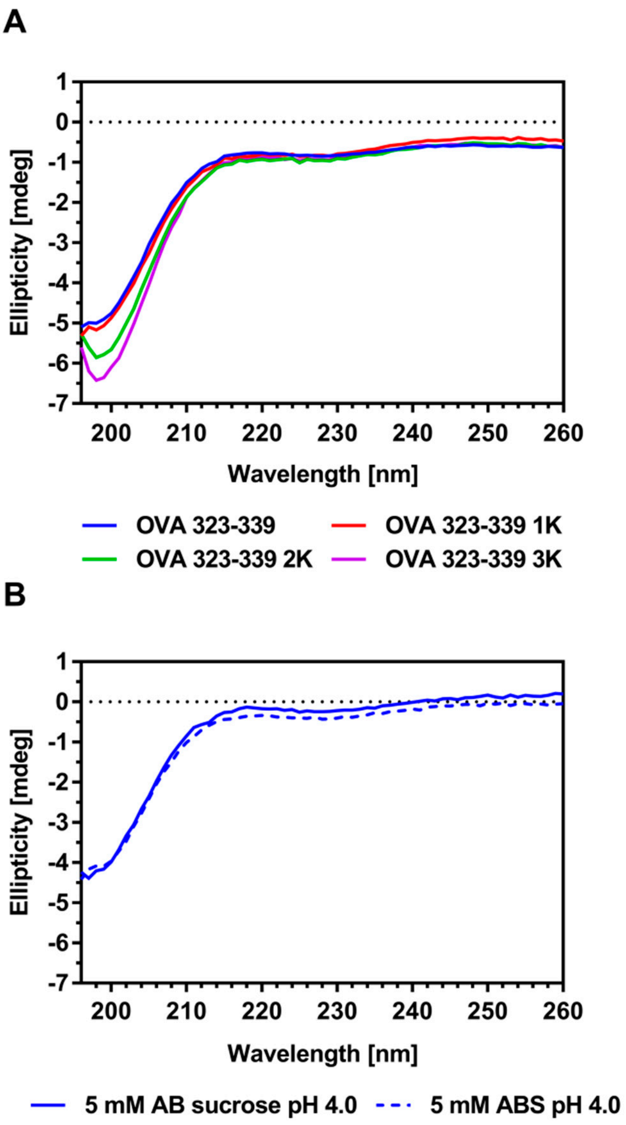

Figure S1. CD spectra of the model T helper cell peptide and its variants. Representative CD spectra of (**A**) OVA 323-339 and its variants in 5 mM PBS pH 6.5 and (**B**) OVA 323-339 in two different acetate buffers at pH 4.0. Measurements were performed at a peptide concentration of 20 µM.

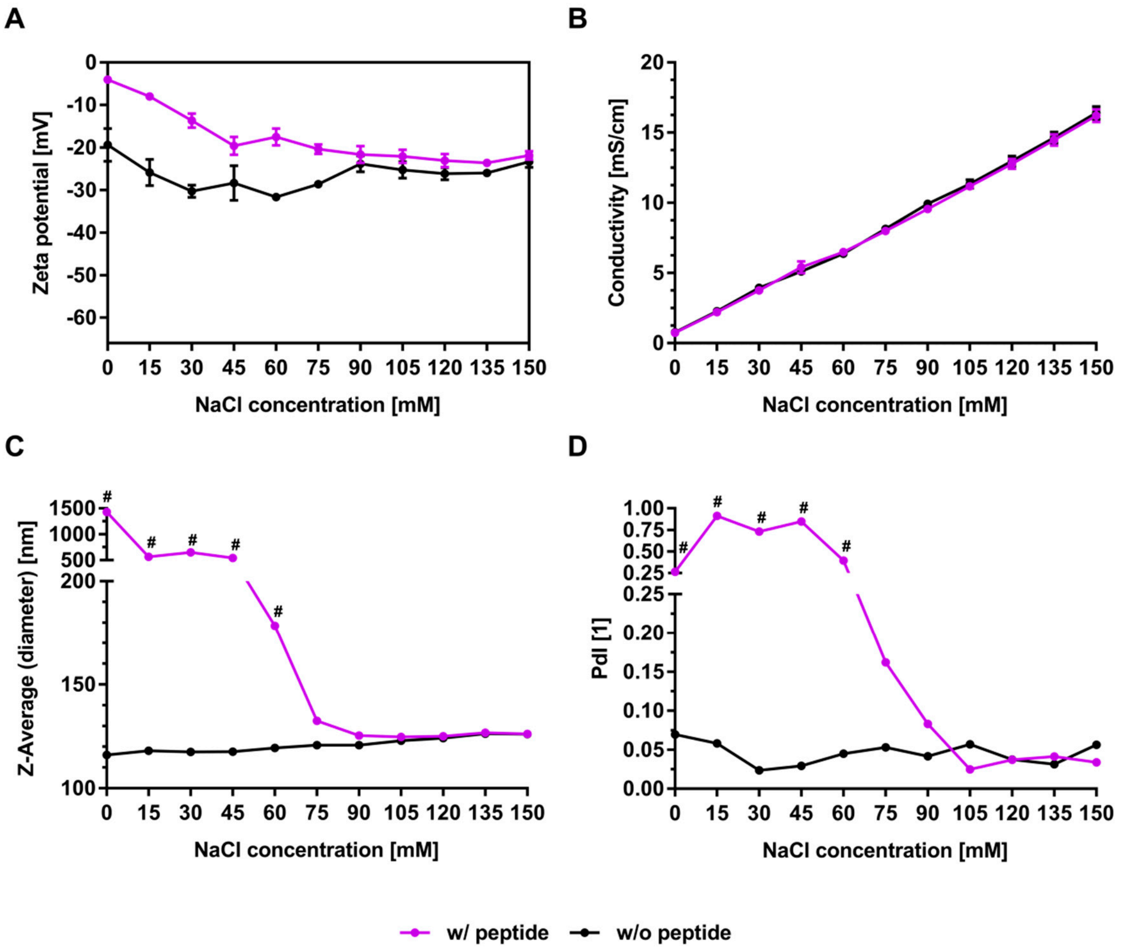

Figure S2. Electrostatic interactions of OVA 323-339 3K with anionic liposomes. (**A**) Zeta potential, (**B**) conductivity, (**C**) Z-Average and (**D**) PdI of liposomes in the presence (w/ peptide) and absence (w/o peptide) of OVA 323-339 3K. All experiments were performed with anionic liposomes in a 5 mM phosphate buffer with a constant pH of 6.5 and varying concentrations of sodium chloride. Experiments w/ peptide were performed at a molar peptide:lipid ratio of 1:133 (50 µM peptide, 6.67 mM lipid). Data represent the mean ± standard deviation from two to three experiments. Hashtags (#) denote particle size analyses of samples that were too polydisperse to fully comply with the requirements of the Zetasizer Nano ZS for a valid measurement.

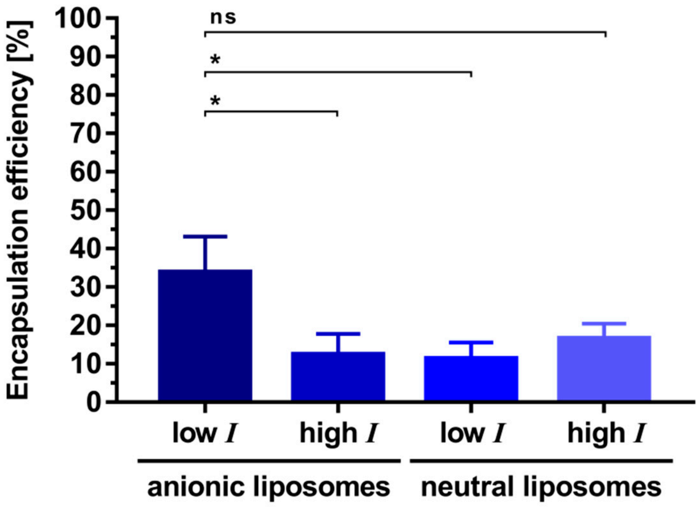

Figure S3. Effect of liposome composition and ionic strength on the encapsulation efficiency of OVA 323-339. Encapsulation of OVA 323-339 at pH 4.0 into liposomes of different composition. Peptide-loaded liposomes were prepared by microfluidic mixing under low- or high-ionic-strength (I) conditions. Data represent the mean ± standard deviation from three experiments. Statistically significant differences are indicated by asterisks: * $P \leq 0.05$ and ns (not significant) $P > 0.05$, using a one-way ANOVA. A correction for multiple comparisons was made using Tukey's test.

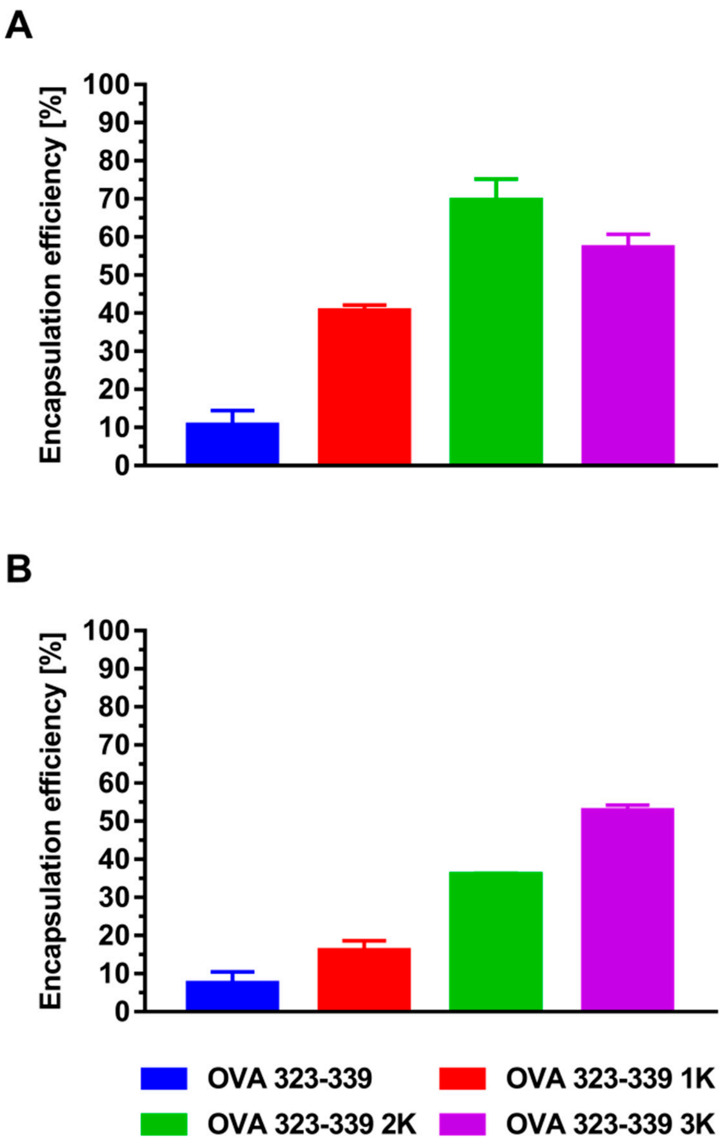

Figure S4. Encapsulation of the model T helper cell peptides into anionic liposomes (non-normalized/non-corrected). Encapsulation efficiency data of peptide-loaded liposomes prepared by (**A**) thin-film hydration or (**B**) microfluidic mixing. Normalized/corrected data are shown in Figure 5. Data represent the mean ± standard deviation from three preparations.

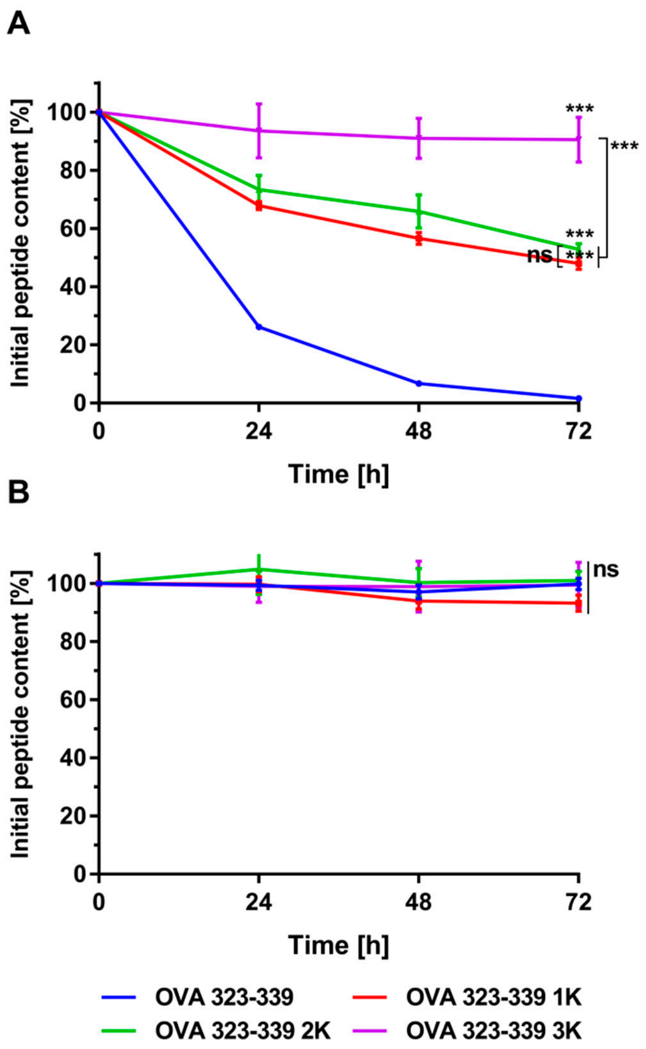

Figure S5. Stability of free and liposomal model T helper cell peptide under cell culture conditions**.** Recovery of (**A**) free and (**B**) liposomal peptides from cell culture medium with serum over a period of 72 h at 37°C. Data represent the mean ± standard deviation from three experiments. Statistically significant differences are indicated by asterisks: *** $P \leq 0.001$ and ns (not significant), using a one-way ANOVA. A correction for multiple comparisons was made using Tukey's test. Unless otherwise indicated, comparisons were always made with the OVA 323-339 experiment at the corresponding time point.

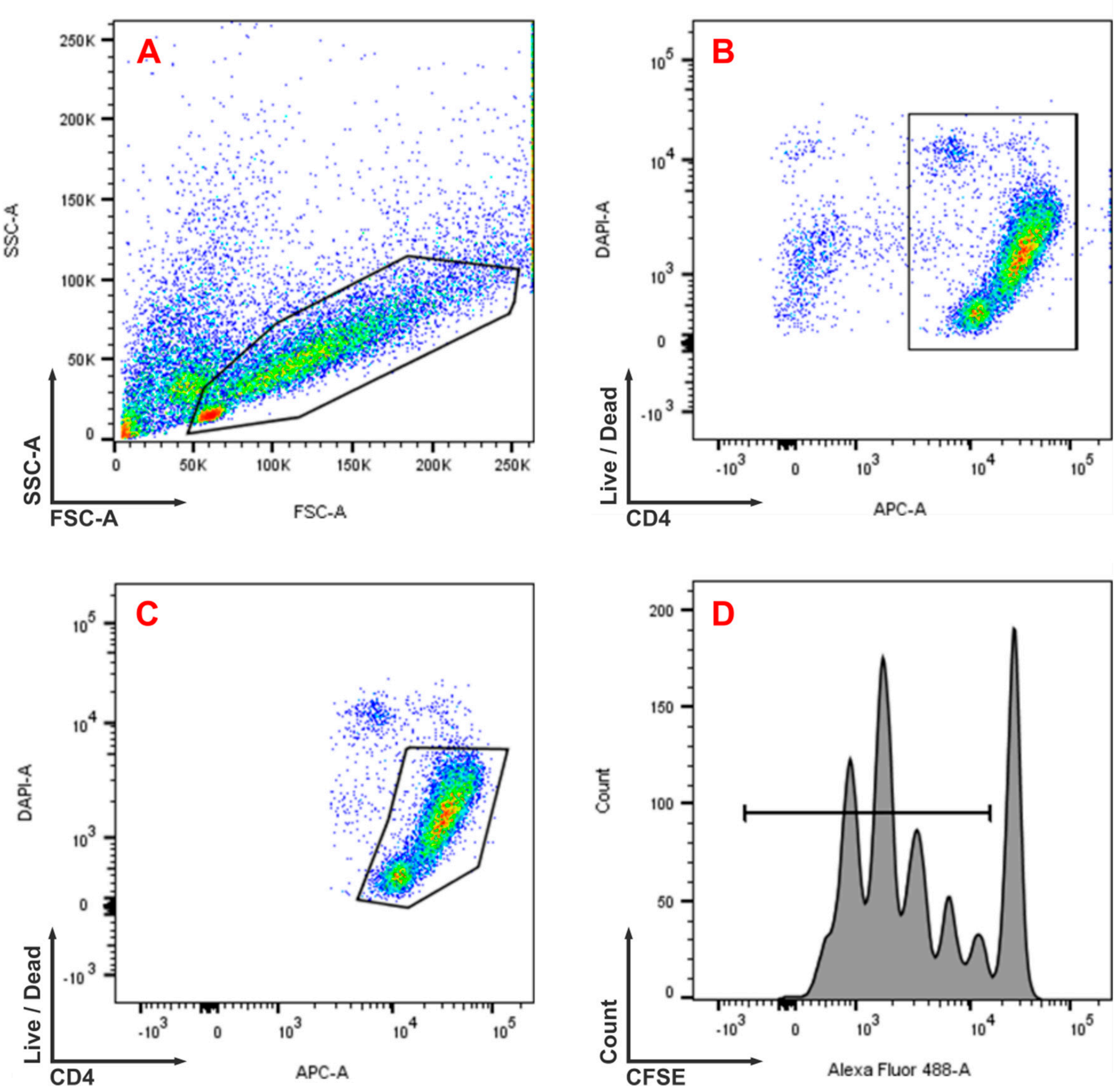

Figure S6. Gating strategy for the experiments shown in Figure 7. (**A**) After gating on lymphoid cells according to their forward and sideward scatter, (**B**) the total CD4⁺ T-cell population was analysed to determine (**C**) the percentage of living cells and (**D**) the percentage of proliferating antigen-specific CD4⁺ T cells (Figure 7A and Figure 7C,D).

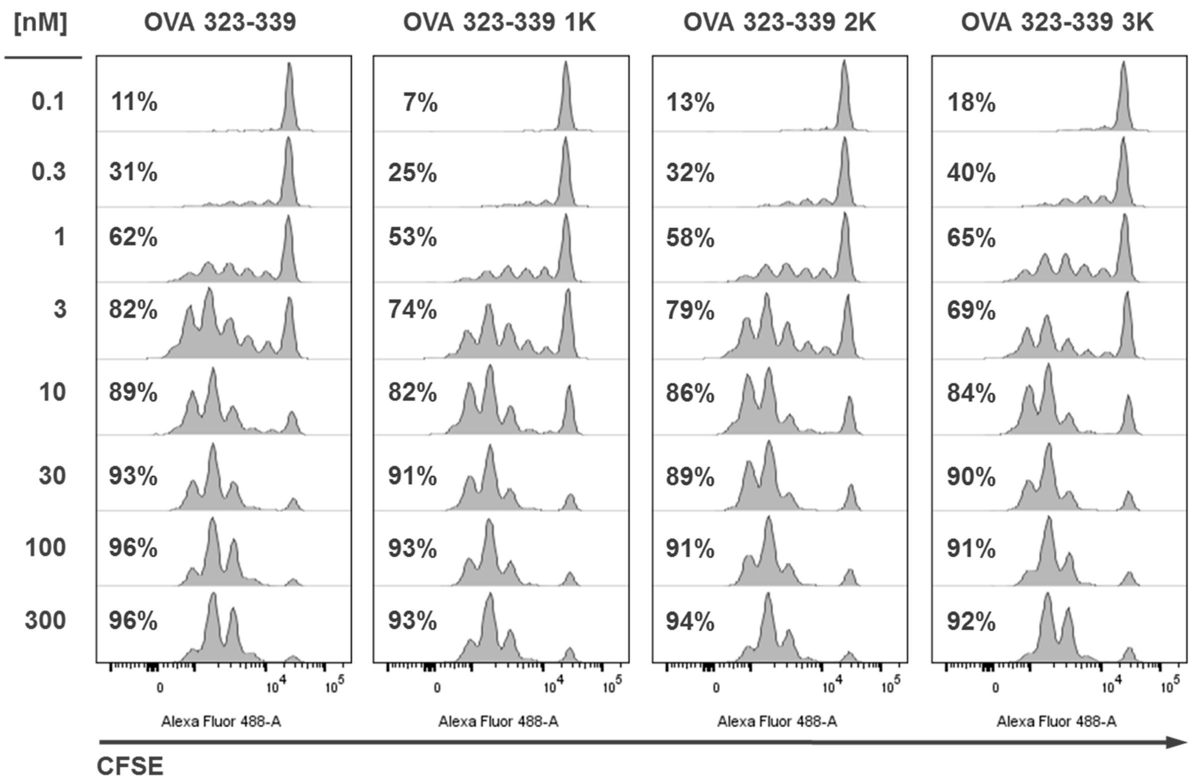

Figure S7. Antigen-specific CD4⁺ T-cell proliferation induced by various free (non-liposomal) OVA 323-339 variants. CD4⁺ T cells were isolated from naïve OT2 mice, labelled with CFSE and co-cultured with splenic DCs from C57BL/6NRj mice for 64 h in the presence of increasing concentrations of peptide. Proliferation of CD4⁺ T cells was measured by means of flow cytometry on the basis of the CFSE dilution patterns (Figure S6). Representative histograms from one of three independent experiments are shown. The full data set was used to calculate the proliferative indices shown in Figure 7.

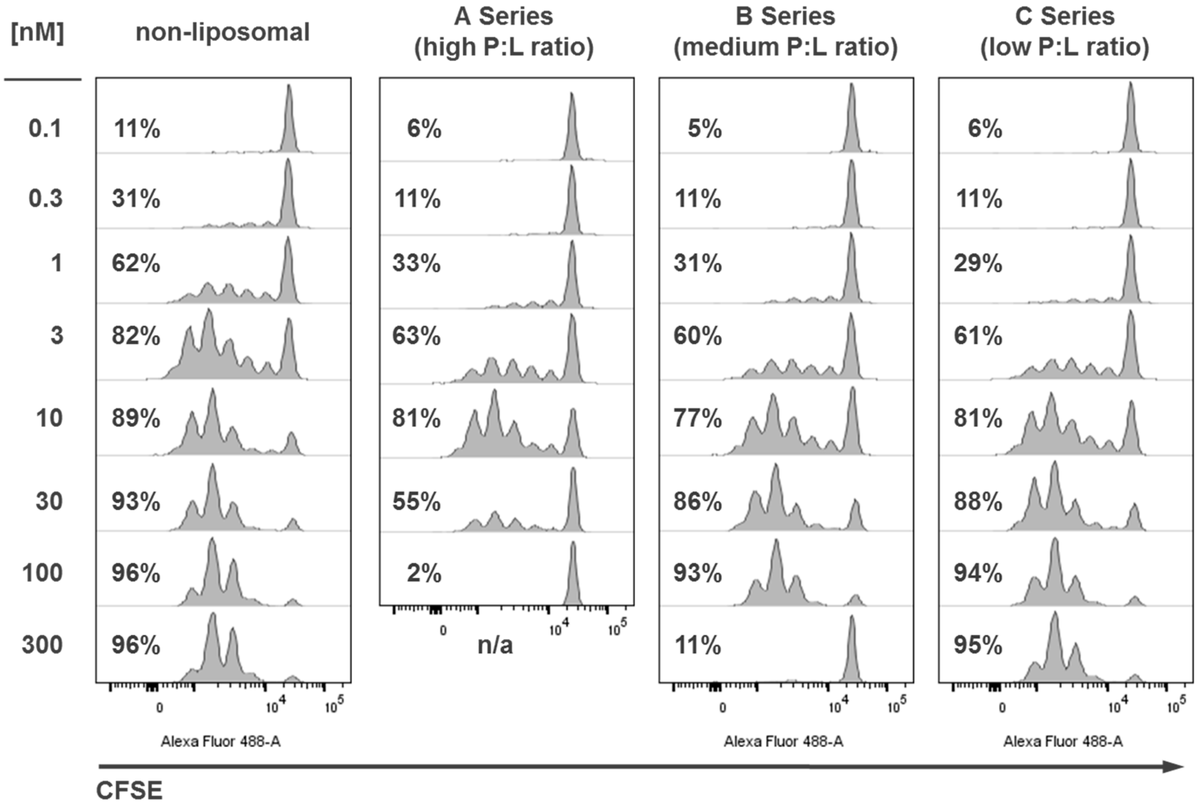

Figure S8. Antigen-specific CD4⁺ T-cell proliferation induced by liposomal OVA 323-339. CD4⁺ T cells were isolated from naïve OT2 mice, labelled with CFSE and co-cultured with splenic DCs from C57BL/6NRj mice for 64 h in the presence of increasing concentrations of peptide. Proliferation of CD4⁺ T cells was measured by means of flow cytometry on the basis of the CFSE dilution patterns (Figure S6). Representative histograms from one of three independent experiments are shown. The full data set was used calculate the proliferative indices shown in Figure 7C,D.
